# Reduced expression of *APC-1B* but not *APC-1A* by the deletion of promoter 1B is responsible for familial adenomatous polyposis

**DOI:** 10.1038/srep26011

**Published:** 2016-05-24

**Authors:** Kiyoshi Yamaguchi, Satoshi Nagayama, Eigo Shimizu, Mitsuhiro Komura, Rui Yamaguchi, Tetsuo Shibuya, Masami Arai, Seira Hatakeyama, Tsuneo Ikenoue, Masashi Ueno, Satoru Miyano, Seiya Imoto, Yoichi Furukawa

**Affiliations:** 1 Division of Clinical Genome Research, Advanced Clinical Research Center, Institute of Medical Science, The University of Tokyo, Tokyo 108-8639, Japan; 2 Gastroenterology Center, Cancer Institute Hospital, Japanese Foundation for Cancer Research, Tokyo 135-8550, Japan; 3 Laboratory of DNA Information Analysis, Human Genome Center, Institute of Medical Science, The University of Tokyo, Tokyo 108-8639, Japan; 4 Laboratory of Sequence Analysis, Human Genome Center, Institute of Medical Science, The University of Tokyo, Tokyo 108-8639, Japan; 5 Department of Clinical Genetic Oncology, Cancer Institute Hospital, Japanese Foundation for Cancer Research, Tokyo 135-8550, Japan; 6 Division of Health Medical Computational Science, Health Intelligence Center, Institute of Medical Science, The University of Tokyo, Tokyo 108-8639, Japan; 7 Division of Health Medical Data Science, Health Intelligence Center, Institute of Medical Science, The University of Tokyo, Tokyo 108-8639, Japan

## Abstract

Germline mutations in the tumor suppressor gene *APC* are associated with familial adenomatous polyposis (FAP). Here we applied whole-genome sequencing (WGS) to the DNA of a sporadic FAP patient in which we did not find any pathological *APC* mutations by direct sequencing. WGS identified a promoter deletion of approximately 10 kb encompassing promoter 1B and exon1B of *APC*. Additional allele-specific expression analysis by deep cDNA sequencing revealed that the deletion reduced the expression of the mutated *APC* allele to as low as 11.2% in the total *APC* transcripts, suggesting that the residual mutant transcripts were driven by other promoter(s). Furthermore, cap analysis of gene expression (CAGE) demonstrated that the deleted promoter 1B region is responsible for the great majority of *APC* transcription in many tissues except the brain. The deletion decreased the transcripts of *APC-1B* to 39–45% in the patient compared to the healthy controls, but it did not decrease those of *APC-1A*. Different deletions including promoter 1B have been reported in FAP patients. Taken together, our results strengthen the evidence that analysis of structural variations in promoter 1B should be considered for the FAP patients whose pathological mutations are not identified by conventional direct sequencing.

Familial adenomatous polyposis (FAP) is an autosomal dominant inherited disorder characterized by the development of hundreds to thousands of colorectal adenomas and subsequent progression to adenocarcinoma. FAP patients also have increased risk of extra-colonic tumors, such as desmoids, osteomas, and tumors in the upper gastrointestinal tract, thyroid, and brain^1^. FAP is caused by a germ line mutation in the tumor suppressor adenomatous polyposis coli (*APC*) gene. The *APC* gene is transcribed into multiple mRNAs of various lengths in a tissue-dependent manner, and NCBI Reference Sequence repository includes three *APC* transcript variants; variant1 (NM_001127511.2), variant2 (NM_001127510.2), and variant3 (NM_000038.5). All transcripts are regulated by either promoter 1A or promoter 1B^2^. These two distinct promoters have been suggested to contribute to the tissue-dependent levels of the transcript variants, but which promoter preferentially functions in each tissue still remains controversial^3^,^4^,^5^.

To date, over 3,800 *APC* variants have been reported on the Leiden Open Variation Database (LOVD)^6^. Evidence from accumulated studies indicates that most of the mutations in *APC* are located in the 5′-half of the gene, and lead to premature truncated protein products due to nonsense mutations or small insertions/deletions. A study of genotype-phenotype correlation demonstrated that the patients with an *APC* mutation in the codon between 1 and 178 or between 312 and 412 show an attenuated form or milder phenotype with later onset and fewer polyps, than the classical polyposis with thousands of polyps in the colon^7^. Although a large number of *APC* germline mutations have been identified by PCR-direct sequencing, there remain a significant number of undiagnosed cases. In these cases, it is considered either that *APC* carries an alteration that is undetectable by the direct sequencing, or alternatively that a pathological genetic change in other genes is involved in the development of tumors reminiscent of FAP. The former cases include patients carrying somatic mosaicism of the *APC* gene with a very low frequency of the mutant allele^8^, and those with structural changes such as large deletions or duplications of *APC*^9^. Recent studies have demonstrated that large deletions of the transcription regulatory region of *APC* result in reduced *APC* transcripts, and that they are involved in a small subset of FAP^4^,^10^,^11^,^12^,^13^,^14^. The latter cases include patients with biallelic germ line mutations in the base excision repair (BER) gene *MUTYH,* an autosomal-recessively inherited disease termed *MUTYH*-associated polyposis (MAP)^15^. In addition, a 40 kb germline duplication upstream of the gremlin 1 (*GREM1*) was identified in patient with hereditary mixed polyposis syndrome^16^. Further studies have uncovered germ line mutations in the proofreading domains of DNA polymerases such as DNA polymerase ε (*POLE*) and δ (*POLD1*) that are associated with adenomatous polyposis and adnocarcinomas in the intestine, which is called polymerase proofreading-associated polyposis (PPAP)^17^. Recently, *NTHL1*, another gene involved in BER, was also shown to be responsible for a recessive type of adenomatous polyposis and carcinoma in the large bowel^18^. It is noteworthy that next-generation sequencing (NGS) has played an important role in the discoveries of new causative gene alterations.

The application of NGS is rapidly expanding from research to clinical practice. We have applied whole-genome sequencing (WGS) for a patient with both multiple adenomatous polyps in the colon and advanced rectal cancer, and without family history of polyposis. This analysis identified a *de novo* deletion in the promoter region of *APC*, which markedly decreased the transcription of the mutant allele. We further disclosed that promoter 1B plays a major role in the expression of *APC*. These data indicate that NGS improves genetic diagnosis of hereditary diseases whose mutations have been overlooked by routine genetic testing, and that analysis of allele specific expression may help the determination of pathological changes in the promoter region of responsible genes.

## Results

### Identification of a large deletion in the promoter region of the *APC* gene

For the genetic diagnosis of the patient, we screened germ line mutations in the *APC* gene by the conventional PCR-direct sequencing method. Although seven variants including six synonymous (rs2229992, rs351771, rs41115, rs42427, rs866006, and rs465899) and one nonsynonymous (rs459552) variants were discovered in the 5′-half of the coding region where most of the *APC* mutations localize, all the seven variants turned out to be non-pathological SNPs by a search in two public databases, LOVD^6^ and ClinVar^19^.

Although multiplex ligation-dependent probe amplification (MLPA) and amplicon sequencing using a panel of disease-associated genes were available for the detection of structural alteration of *APC* and genetic alterations in other responsible genes, respectively, we took advantage of WGS to test its potential in clinical genetic testing. Thus, WGS was performed using DNA extracted from the patient’s peripheral blood to explore pathological mutation(s) associated with the disease. An average coverage depth of nearly 30× was achieved, and 19,660 variants and 299 short insertion-deletion variants were detected in exons or splice sites. First, we searched for deleterious variants in the genes associated with adenomatous polyposis of the colon (*APC, MUTYH, NTHL1, POLD1*, and *POLE*). In complete agreement with the data obtained by the conventional screening, we confirmed the seven non-pathological mutations by WGS and failed to identify additional mutations or small insertions/deletions in *APC*. Evaluation of the non-synonymous SNVs disclosed that *MUTYH* p.Q338H (rs3219489) is a non-pathological variant, and that *NTHL1* p.L301V (rs200420874) is a variant of uncertain significance (VUS) (Table 1). Although we additionally searched for germ line mutations in other genes that predispose to colorectal polyps such as *SMAD4* and *BMPR1A* (juvenile polyposis syndrome), *STK11* (Peutz-Jeghers syndrome), *GREM1* (hereditary mixed polyposis syndrome), and *PTEN* (Cowden syndrome), all variants in these genes were not deleterious but non-pathological SNPs (Supplementary Table S1).

**Figure 1:**
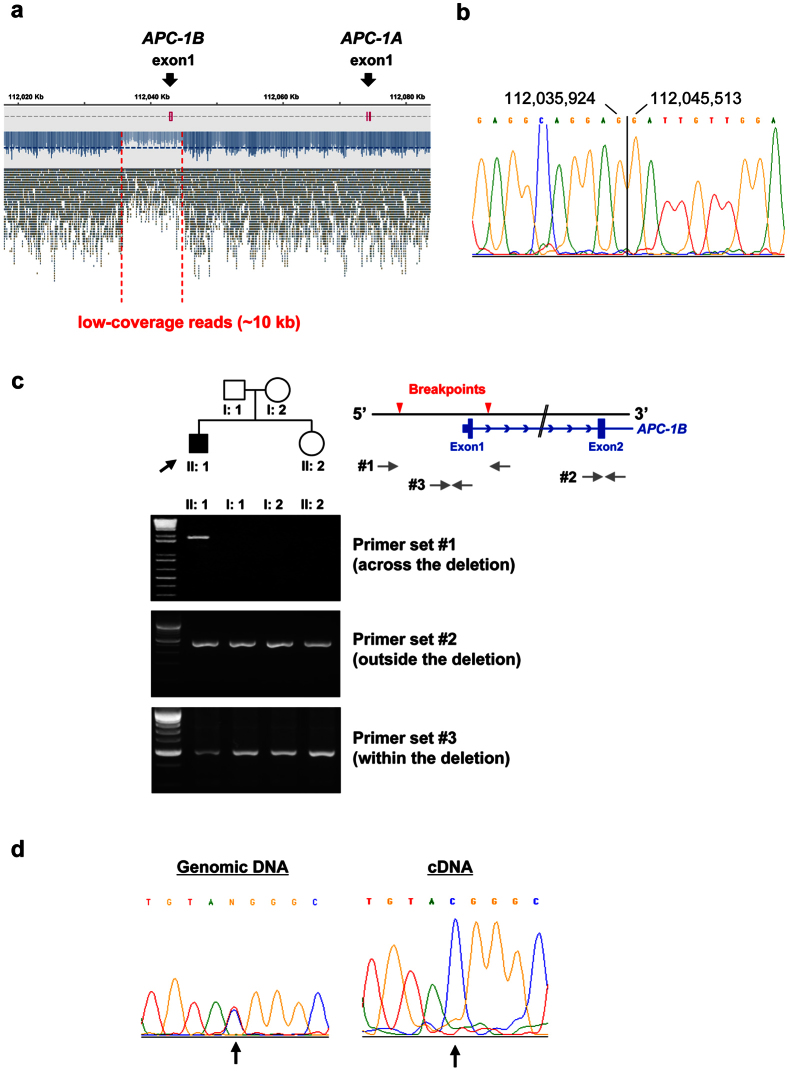
(**a**) A snapshot of the Tablet, a graphical viewer for NGS assemblies and alignments, showing a large deletion of ~10 kb encompassing promoter 1B and exon1B in the *APC* gene. (**b**) Direct sequencing of the breakpoint region. The PCR amplification was performed using primers across the deleted region (Supplementary Table S3), followed by the Sanger sequencing of the amplified fragment. (**c**) The PCR analysis for the deletion in the first-degree relatives. Three sets of primers were used for the amplification of a region across the deletion (upper panel), *APC-1B* exon2 (NM_001127511.2, middle panel), and a region within the deletion (lower panel). The PCR amplification of *APC-1B* exon2 was used as an internal control. The primer sequences are shown in Supplementary Table S3. The proband is indicated by an arrow. Unaffected and affected individuals are represented by open and closed symbols (square: male and circle: female), respectively. (**d**) The sequence analysis of the heterozygous SNP rs2229992 using genomic DNA and cDNA. The arrow indicates the position of rs2229992 (c.1458 T/C).

To determine structural alterations such as large insertions/deletions that account for the disease, we analyzed copy number alteration in genes and the flanking regions associated with adenomatous polyposis. Consequently, a significant copy number loss was found in the *APC* gene (Supplementary Fig. S1a). A visualization of the aligned reads exhibited the 5′-flanking region of *APC* with decreased sequence reads as shown in Fig. 1a, suggesting a large deletion encompassing the regulatory region (known as promoter 1B) and exon1 of the *APC* variant1 (*APC-1B*). Subsequent PCR analysis using primers across the deleted region successfully amplified the mutated allele, and the breakpoint of deletion was determined by Sanger sequencing of the PCR products (Fig. 1b). The size of the deletion was 9,588 bp (chr5:112, 035, 925–112, 045, 512; GRCh37/hg19). A homology search revealed that the flanking sequences of the breakpoints shared 77.5% identity across 200 bp (Supplementary Fig. S1b). Interestingly, the breakpoints are located within Alu repeat elements, suggesting that the deletion was likely caused by Alu-mediated recombination. The deletion of promoter 1B was confirmed by MLPA including probes for detecting two *APC* promoters (Supplementary Fig. S2a). Although deletions of promoter 1B have been reported by other groups^4^,^11^,^12^,^13^,^20^, this is the first report of a promoter deletion of *APC* in Japanese patients. To examine the inheritance of disease, we investigated the deletion in the first-grade relatives of the patient. PCR analysis using a set of primers across the deletion successfully amplified the genomic region encompassing the deletion in the patient. However, we failed to amplify the region with genomic DNA of the relatives (Fig. 1c, upper panel). In addition, semi-quantitative PCR analysis using a set of primers designed within the deletion showed that the PCR products of the patient were reduced compared with the relatives (Fig. 1c, lower panel). On the other hand, the amount of PCR products with a set of primers outside the deletion was unchanged between the patient and the relatives (Fig. 1c, middle panel). These data suggest that the deletion is not carried in the relatives, indicating a *de novo* mutation in the patient. It is of note that this result is compatible with the patient’s family history because none of the relatives were diagnosed as colonic polyposis, and no polyposis was detected in the first-grade relatives by colonoscopy.

### Transcriptional repression of the mutant *APC* allele

Since the patient carried a large deletion which included promoter 1B, the mutant *APC* allele should have reduced transcriptional activity. Previous reports demonstrated that deletion of promoter 1B substantially reduced *APC* transcripts^4^,^10^,^11^,^12^,^13^,^14^. Based on this view, we speculated that one of the two alleles is down-regulated in the peripheral leucocytes as well as the epithelial cells of the colonic mucosa. To analyze allele-specific expression (ASE) of *APC* in the patient, we took the advantage of the heterozygous SNP (rs2229992) in exon12 (NM_000038.5), and performed Sanger sequencing of the PCR products encompassing the SNP. This analysis indicated that both T and C alleles were retained in the genomic DNA (Fig. 1d). However, the T allele had almost disappeared in the cDNA, suggesting that the mutant transcripts were completely lost in the peripheral leucocytes. Due to the technical limitations of detection sensitivity in Sanger sequencing (less than 15%)^8^,^21^, we further performed deep sequencing of the cDNA, which enabled us to estimate the ratio of the two alleles in the transcripts by counting the sequence reads of the PCR products. The number of reads on the T allele was extremely low compared to the C allele (3,191 vs 25,246 reads). It is rational to consider that the T and C alleles correspond to mutant and wild type alleles, respectively. This view lead us to speculate that the 3,191 sequence reads of T allele may mainly consist of residual *APC-1A* transcript, and that the 25,246 reads of C allele include both *APC-1A* and *APC-1B* transcripts. We estimated that the mutant transcripts make up only 11.2% (3,191/28,437) of the total *APC* transcripts.

### Transcription of the mutant allele starts from the second TSS by promoter 1A

**Figure 2:**
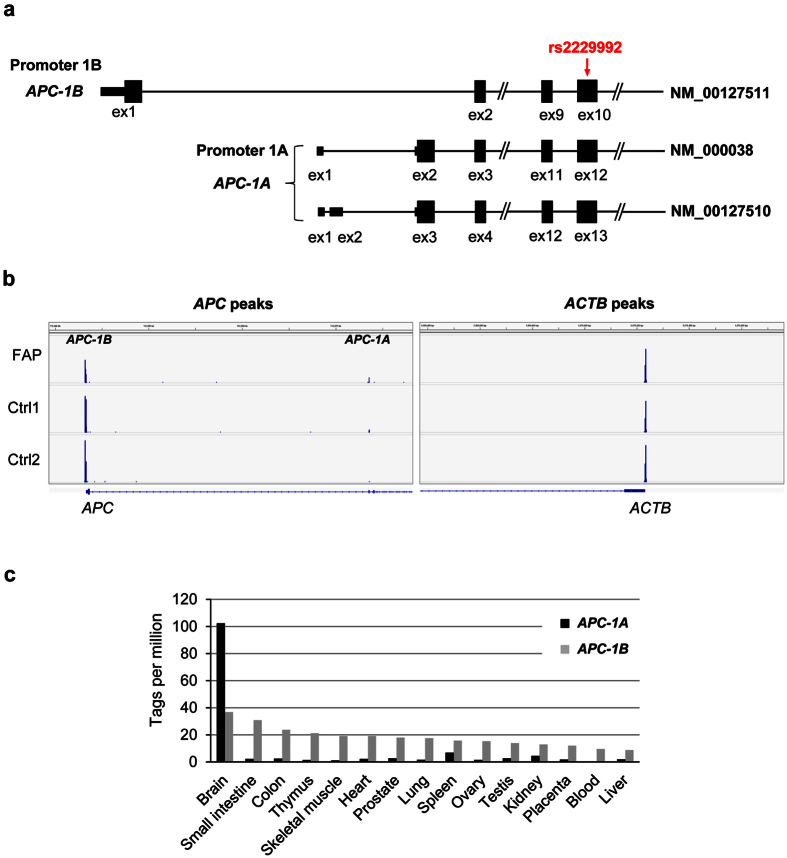
(**a**) Schematic diagram of human *APC* transcript variants listed in NCBI. *APC* exhibits two forms of transcripts from promoter 1A and 1B, defined as *APC-1A* and *APC-1B*, respectively. The arrow indicates the position of heterozygous SNP rs2229992 (c.1458T/C). (**b**) The Integrative Genomics Viewer (IGV) snapshot of the three CAGE experiments, showing the *APC-1A, APC-1B*, and *ACTB* peaks. The *APC-1B* peak in the FAP patient was much lower than those in controls. The numbers of mapped reads within each peak are shown in Table 2. (**c**) Organ/tissue-dependent expression of *APC-1A* and *APC-1B* were identified using a CAGE-based expression data from the FANTOM5 SSTAR (http://fantom.gsc.riken.jp/5/sstar/Main_Page).

The *APC* gene has two promoter regions (Fig. 2a), and promoter 1B is located approximately 30 kb upstream of promoter 1A. Although the mutant allele lacks the region encompassing promoter 1B and exon1B, it retains promoter 1A. To investigate the activity of promoter 1A and/or others on the mutant allele, we employed cap analysis of gene expression (CAGE) analysis that allows transcriptome profiling with the simultaneous identification of tissue/cell/condition-dependent transcriptional start site (TSS). This analysis facilitates the estimation of the *APC* promoter activity by the measurement of peak height at the TSS. RNAs used for this analysis were extracted from the peripheral blood of two healthy volunteers as well as from the patient. Expectedly, we identified two tag clusters (Fig. 2b); the first cluster located at the 3′ site of promoter 1B, and the second cluster at the 3′ site of promoter 1A. The RNA from the two healthy volunteers showed both these peaks, and the first peak at 1B was approximately 44.5-fold higher than the second peak at 1A. Importantly, the peak at 1B was reduced in the patient to approximately 50% (39–45%) compared to the peak in the healthy volunteers (control 1 and control 2), while the peaks in *ACTB* showed no significant difference between the two (Table 2). Considering the expression of the wild type allele in the patient, the CAGE data suggested a complete loss of promoter 1B activity in the mutant allele. Of note, the CAGE data detected the peak at promoter 1A in the patient, but no new substantial peaks appeared in the flanking region, suggesting that the activity of promoter 1A was not abrogated by the deletion. In addition, the peak was slightly increased in the patient compared to the healthy controls (Fig. 2b).

Degradation of cytosolic β-catenin by the *APC* destruction complex represents the key step of the canonical Wnt signaling pathway. The increased stability of β-catenin protein following *APC* mutation leads to the transcriptional activation of target genes in the nucleus^22^. Although we estimated approximately a 50% reduction of *APC* transcripts in the patient, we did not find any significant up-regulation of Wnt-target genes such as *DKK1, MYC*, and *CCND1* (Table 3). Since *APC* is a tumor suppressor gene, complete inactivation of the protein may be necessary for the transcriptional upregulation of these downstream genes. Consistently, motif discovery analysis showed that T-cell factor/lymphoid enhancer factor (TCF/LEF) motifs were not significantly enriched in the up-regulated gene list (Supplementary Data S1).

### Transcription start sites of *APC* in various normal tissues

To elucidate the levels of *APC* expression in different tissues, we leveraged a CAGE-based gene expression database (http://fantom.gsc.riken.jp/5/sstar/Main_Page). The data is useful for the measurement of relative levels of transcripts initiated at each TSS among different tissues because CAGE does not require any intervening amplification steps and uses simple normalization. The levels of *APC-1A* and *APC-1B* expression in the tissues are listed in Supplementary Table S2. Consistent with our results and a previous report^4^, the expression of *APC-1B* was much higher than that of *APC-1A* in the blood, implying that promoter 1B plays a major role in the regulation of *APC* expression in most normal tissues (Fig. 2c).

## Discussion

Not only point mutations and small insertions/deletions but also structural alterations in the genome play a crucial role in human diseases. Large deletions/amplifications have been shown to be responsible for familial cancer syndromes such as hereditary breast and ovarian cancer^23^,^24^, FAP^25^, and Lynch syndrome^26^. MLPA is available to capture large genomic deletions/amplifications in the responsible genes. Regarding FAP, Aretz *et al*. screened deletions/amplifications of *APC* in 174 unsolved cases by MLPA, and identified 14 different types of deletions in 26 cases, ranging from a single exon to the whole gene including the regulatory region^9^. Genetic testing by direct sequencing followed by MLPA analysis has increased the detection rate of pathogenic mutations in *APC* by 7–10%^11^. However, the identification of genetic alterations in the 5′-flanking regions, the intronic regions, and the 3′-untranslated regions of the responsible genes remains a challenge in genetic testing^27^.

A limited number of articles report FAP cases with promoter 1B deletion^4^,^10^,^11^,^12^,^13^,^14^,^20^. These cases included deletions with different sizes ranging from 11 kb to 132 kb (Supplementary Fig. S2b). The patient in this study carried a deletion of ~10 kb, which is the smallest deletion of promoter 1B, known to date. It is of note that these cases including ours did not show the phenotype of attenuated FAP but that of classical FAP. Since the germ line deletion encompassing the promoter 1B resulted in a marked decrease of *APC* transcripts in spite of the remaining *APC-1A* transcript, deletions or point mutations within this ~10 kb region may be involved in FAP without mutations in the coding region.

Although deletions of promoter 1B have been shown to reduce the expression of mutant *APC* allele, the degree of reduction caused by the deletions is different among previous reports. Pavicic *et al*. estimated the ASE by single nucleotide primer extension analysis, and found that a large deletion of 132 kb resulted in a reduction of the deleterious allele expression to 40–60% of the wild-type allele^13^. Another report revealed that a deletion of ~61 kb led to approximately a 90% reduction^4^. In this study, we used two approaches, ASE analysis with deep sequencing and CAGE, and disclosed that the deletion caused 87% reduction of mutant allele expression. Interestingly, the residual transcription was maintained by promoter 1A although the activity of promoter 1B was completely lost. Of note, the expression of *APC-1A* was slightly higher in the mutation carrier than that in controls. This may be explained as a compensatory mechanism against the impaired activity of promoter 1B. A similar observation has been reported by Rohlin *et al*.^4^.

To our knowledge, this is the first report showing a tissue-specific regulation of the *APC* transcripts at the TSS levels. We here revealed in our CAGE analysis that *APC-1B* expression is much higher than *APC-1A* expression in leukocytes, which is in complete accordance with the CAGE-based expression data in FANTOM5 SSTAR (Fig. 2b,c). Since CAGE does not require any intervening amplification step and uses simple normalization, it is suitable for measuring relative levels of transcripts initiated at each TSS among different tissues. Of note, the brain expresses an extremely high amount of *APC-1A* in addition to the high levels of *APC-1B* expression. This finding may suggest an important role of *APC* in the brain. It has been reported that canonical Wnt signaling pathway is one of the essential regulators of adult neurogenesis^28^,^29^,^30^. Strikingly, the expression of *APC* plays a key role in regulating neuronal differentiation of newly generated cells and maintaining neural stem cells in the adult neurogenic niche^31^. Although the role and mechanisms(s) of abundant *APC-1A* expression in the brain remain unclear, we speculate that patients lacking promoter 1B may not suffer from brain tumors associated with FAP.

In conclusion, using next-generation sequencing we identified a new causative promoter deletion of *APC* at the genome and transcript levels in a Japanese FAP patient. Of note, whole-genome sequencing in combination with ASE analysis by deep sequencing may be a useful strategy to identify deleterious genetic alterations in the regulatory regions of responsible genes for hereditary diseases.

## Materials and Methods

### Patient and DNA/RNA extraction

A 29-year-old man was diagnosed to suffer from multiple colon adenomas and concomitant rectal adenocarcinoma by colonoscopy (Supplementary Fig. S2c,d). Additional upper gastrointestinal endoscopy revealed multiple fundic gland polyps in the stomach, and adenomas in the duodenum. These clinicopathological findings strongly suggested classical FAP. The patient’s family tree is shown in Supplementary Fig. S3. None of the other family members were diagnosed as colonic polyposis. Individuals, I-1 and I-2 suffered gastric cancer (GS) and colorectal cancer (CRC), respectively. Individual, I-4 suffered CRC and thyroid cancer (TC). However, their offspring has had no malignancies except for the patient. The first-grade relatives of the patient (II-3, 4, and III-5) underwent screening of the colon by colonoscopy, but no polyposis was detected at the age indicated in Supplementary Fig. S3.

Genomic DNA was extracted from peripheral blood of the patient according to the standard phenol extraction/purification procedure^32^. Peripheral blood mononuclear cells were isolated from peripheral blood using Ficoll stratification. Total RNA was extracted using RNeasy Mini Kit (Qiagen, Hilden, Germany).

This project was approved by the ethical committee of Institute of Medical Science, the University of Tokyo (IMSUT-IRB, 23-18-0929). All experiments were carried out in accordance with the approved guidelines. Written informed consent was obtained from the patient and the first-degree relatives in this study.

### PCR-direct sequencing

The coding exons in *APC* were amplified with M13-tailed target-specific primers, and the PCR products were sequenced on the Applied Biosystems 3730xl DNA Analyzer using the BigDye Direct Cycle Sequencing Kit (Thermo Fisher Scientific, Waltham, MA). The primer sequences used for sequencing are available on request.

### Whole-genome sequencing

WGS was performed using the HiSeq 2500 platform with paired-end reads of 101 bp according to the manufacturer’s instructions (Illumina, San Diego, CA). A sequence library containing inserts of 250–350 bp was prepared using 1.0 μg of genomic DNA extracted from lymphocytes. A fastq file was aligned to human reference sequence (hg19) using Burrows-Wheeler Aligner (ver. 0.5.10)^33^ and a bam file was created for data processing. The detection of single nucleotide variants and short insertions/deletions was performed by comparing the aligned bases with the reference sequence using a Bayesian approach. At each candidate position, the posterior probability of existence of variant was computed by updating a beta non-informative prior with the information of observed reads. We supposed that a random variable ‘X’ follows the posterior distribution representing the probability of variant. We used Pr(X ≥ 0.05) as the score, and regarded the candidate positions whose scores were greater than 0.9 as statistically significant. Pathogenicity of the variants was first evaluated by two public databases, Leiden Open Source Variation Database (LOVD) and ClinVar in National Center for Biotechnology Information (NCBI). Uncharacterized non-synonymous variants were secondary evaluated by the frequencies of mutant alleles using dbSNP in NCBI, ESP-6500 in NHLBI Exome Sequencing Project, Human Genetic Variation Database (HGVD) in Kyoto University, and Integrative Japanese Genome Variation Database (iJGVD) in Tohoku University. Variants in *APC, BMPR1A*, or *STK11* with an allele frequency greater than 0.01, and those in *MUTYH* or *NTHL1* greater than 0.05 were considered as non-pathologic variants. The remaining non-synonymous variants were categorized in VUS. Additionally, a copy number analysis was performed using WGS data. Abnormal copy number regions were detected using the circular binary segmentation algorithm with the R package DNAcopy^34^.

### Determination of the breakpoint and analysis of allele-specific expression of the *APC* gene

The breakpoint of the deletion was determined by PCR amplification using primers across the deleted region, followed by the Sanger sequencing of the amplified fragment. The primer sequences used for the amplification are shown in Supplementary Table S3.

To examine allele-specific expression (ASE) of *APC*, deep cDNA sequencing was performed using IonPGM Sequencing 200 kit and Sequencing 200 kit (Thermo Fisher Scientific) with libraries of PCR products prepared using Ion Plus Fragment Library Kit (Thermo Fisher Scientific). One microgram of total RNA was reverse transcribed for single-stranded cDNA using oligo(dT)_18_ primer with Transcriptor reverse transcriptase (Roche Diagnostics, Indianapolis, IN). The cDNA was amplified with a set of primers encompassing a heterozygous single nucleotide polymorphism (SNP) at rs2229992 in the coding region of *APC*. The primer sequences are shown in Supplementary Table S3. Variants were identified using the Variant Caller deployed with Torrent Suite (Thermo Fisher Scientific), and the ASE was estimated using the allele-specific read depth.

### Identification of a *de novo APC* mutation in the patient

PCR amplification was performed using DNA from the patient and the first-degree relatives with a set of primers across the deletion. Additionally, semi-quantitative PCR was performed with a set of primers within the deleted region and those outside the deletion (KOD-Plus kit, TOYOBO, Osaka, Japan). The primer sequences are shown in Supplementary Table S3.

### Cap analysis of gene expression (CAGE)

To elucidate relative levels of transcripts initiated at each TSS, we employed CAGE, a promoter-based expression analysis. The CAGE cDNA libraries were prepared using a Library Preparation kit according to the manufacturer’s protocol (DNAFORM, Yokohama, Japan). Briefly, two micrograms of total RNA extracted from peripheral blood were used for synthesis of the first strand cDNA. After biotinylation of 5′-caps of RNAs, these biotinylated RNA/cDNA hybrids were captured by streptavidin-conjugated magnetic beads. The cDNAs were released from RNAs, and ligated with a linker including barcode sequences. Sequencing of the CAGE libraries were performed on the Illumina HiSeq 2500 (50 bp single-end). The CAGE sequencing data were handled using Maser (Management and Analysis System for Enormous Reads), a pipeline execution system, provided by the National Institute of Genetics (http://cell-innovation.nig.ac.jp/index_en.html). CAGE tags were normalized by the total number of tags per sample mapped to the human genome (hg19), and were indicated as tags per million (TPM). The clusters with <0.1 TPM per base were omitted. To compare the levels of *APC* expression in different normal human tissues, we used FANTOM5 SSTAR (semantic catalog of samples, transcription initiation and regulators), a CAGE-based gene expression database, provided by Riken (http://fantom.gsc.riken.jp/5/sstar/Main_Page).

## Additional Information

**How to cite this article**: Yamaguchi, K. *et al*. Reduced expression of *APC-1B* but not *APC-1A* by the deletion of promoter 1B is responsible for familial adenomatous polyposis. *Sci. Rep.* **6**, 26011; doi: 10.1038/srep26011 (2016).

## Supplementary Material

Supplementary Information

## Figures and Tables

**Table 1:** Summary of variants in genes associated with adenomatous polyposis of the colon (*APC, MUTYH, NTHL1, POLD1*, and *POLE*) detected by WGS.

Gene	Type	Mutation	Protein alteration	dbSNP
*APC*	synonymous	c.T1458C	p.Y486Y	rs2229992
*APC*	synonymous	c.G1635A	p.A545A	rs351771
*APC*	synonymous	c.G4479A	p.T1493T	rs41115
*APC*	synonymous	c.G5034A	p.G1678G	rs42427
*APC*	synonymous	c.T5268G	p.S1756S	rs866006
*APC*	nonsynonymous	c.T5465A	p.V1822D	rs459552
*APC*	synonymous	c.G5880A	p.P1960P	rs465899
*MUTYH*	nonsynonymous	c.G1014C	p.Q338H	rs3219489
*NTHL1*	nonsynonymous	c.C901G	p.L301V	rs200420874
*POLE*	synonymous	c.G1323A	p.P441P	rs116573514
*POLE*	synonymous	c.C2935T	p.L979L	rs56081968
*POLE*	synonymous	c.G3156A	p.T1052T	rs5744857
*POLE*	synonymous	c.A4530G	p.A1510A	rs5744944
*POLE*	synonymous	c.C5334T	p.A1778A	rs11146986
*POLE*	synonymous	c.A6252G	p.S2084S	rs5745022

**Table 2:** The expression of *APC* and *ACTB* in the patient and controls analyzed by CAGE.

Gene	Patient	Control 1	Control 2
*APC-1B*	11.70	30.28	26.18
*APC-1A*	1.72	0.68	<0.1
*ACTB*	2526.07	2208.45	2742.55

Each value represents CAGE tags that are normalized as TPM (tags per million).

*ACTB* served as a control.

**Table 3:** The expression of Wnt target genes in the patient and controls analyzed by CAGE.

Gene	Patient	Control 1	Control 2
*DKK1*	0.57	0.68	0.61
*MYC*	31.31	59.99	46.75
*CCND1*	0.34	0.91	0.97
*PPARD*	44.38	40.37	40.54
*AXIN2*	4.36	7.14	7.55
*LGR5*	<0.10	< 0.10	0.12
*CD44*	780.90	573.60	784.35

Each value represents CAGE tags that are normalized as TPM (tags per million).
