# Supplementary material for: Reduced expression of APC-1B but not APC-1A by the deletion of promoter 1B is responsible for familial adenomatous polyposis
Source: Sci Rep. 2016 May 24;6:26011. doi: 10.1038/srep26011 (PMC4877598; doi:10.1038/srep26011)
Supplement: Supplementary Information [file srep26011-s1.pdf]

Reduced expression of *APC-1B* but not *APC-1A* by the deletion of promoter 1B is responsible for familial adenomatous polyposis

Kiyoshi Yamaguchi, Satoshi Nagayama, Eigo Shimizu, Mitsuhiro Komura, Rui Yamaguchi, Tetsuo Shibuya, Masami Arai, Seira Hatakeyama, Tsuneo Ikenoue, Masashi Ueno, Satoru Miyano, Seiya Imoto, Yoichi Furukawa

Supplementary Tables S1, S2, and S3

Supplementary Figures S1, S2, and S3

Supplementary Data S1

Supplementary Table S1 Summary of variations in additional polyposis syndrome-associated gene detected by WGS

Gene	Type	Mutation	Protein alteration	dbSNP
BMPR1A	nonsynonymous	c.C4A	p.P2T	rs11528010
GREM1	synonymous	c.C207G	p.P69P	rs2280738
STK11	nonsynonymous	c.C1062G	p.F354L	rs59912467

Five high-penetrance genes that predisepose to colorectal polyps; *SMAD4* and *BMPR1A* (juvenile polyposis syndrome), *STK11* (Peutz-Jeghers syndrome), *GREM1* (hereditary mixed polyposis syndrome), and *PTEN* (PTEN hamartoma syndrome).

Supplementary Table S2 Organ/tissue-dependent expression of *APC-1A* and *APC-1B*

Samples*	1A#	1B#	1B/1A
brain, adult, pool1.CNhs10617.10012-101C3	102.32	36.83	0.36
small intestine, adult, pool1.CNhs10630.10024-101D6	2.24	30.87	13.78
colon, adult, pool1.CNhs10619.10014-101C5	2.48	23.71	9.56
thymus, adult, pool1.CNhs10633.10027-101D9	1.42	21.19	14.92
skeletal muscle, adult, pool1.CNhs10629.10023-101D5	1.13	19.19	16.98
heart, adult, pool1.CNhs10621.10016-101C7	2.23	19.19	8.61
prostate, adult, pool1.CNhs10628.10022-101D4	2.67	17.97	6.73
lung, adult, pool1.CNhs10625.10019-101D1	1.6	17.51	10.94
spleen, adult, pool1.CNhs10631.10025-101D7	6.86	15.69	2.29
ovary, adult, pool1.CNhs10626.10020-101D2	1.5	15.27	10.18
testis, adult, pool1.CNhs10632.10026-101D8	2.65	13.94	5.26
kidney, adult, pool1.CNhs10622.10017-101C8	4.49	12.92	2.88
placenta, adult, pool1.CNhs10627.10021-101D3	1.77	12.06	6.81
blood, adult, pool1.CNhs11761.10053-101G8	<0.1	9.58	-
liver, adult, pool1.CNhs10624.10018-101C9	1.91	8.79	4.60

*FANTOM5 SSTAR provides detailed sample information (http://fantom.gsc.riken.jp/5/sstar/Browse_samples).

#Values are indicated as tags per million (TPM).

Supplementary Table S3 Primer sequences used for PCR

Primer	Strand	Sequence (5'-3')	Application purpose
Spanning SNP (rs2229992)	F	AATGAACTAGGGGGACTACAG	cDNA sequencing to identify allele-specific expression of *APC*
	R	AAGTTTGTCAAAGCCATTCCAG	
Spanning the deletion	F	CAACAAGTTACATAGCATCTATCCTG	Mapping promoter 1B deletion breakpoints and deletion analysis
	R	GATCATGGGATCCTGGTTACAC	
APC-1B exon2 (NM_001127511.2)	F	TGTAAAACGACGGCCAGTTTTCAGTCATGTATATTTGTGG	Internal control for deletion analysis
	R	CAGGAAACAGCTATGACCTAAGTGTTAGCTATCACCTAC	
Within the deletion	F	CCAGGAGCCTTGGATGGTTAG	Deletion analysis
	R	GAATTCTAAGACGTCAGTAGGC	

a

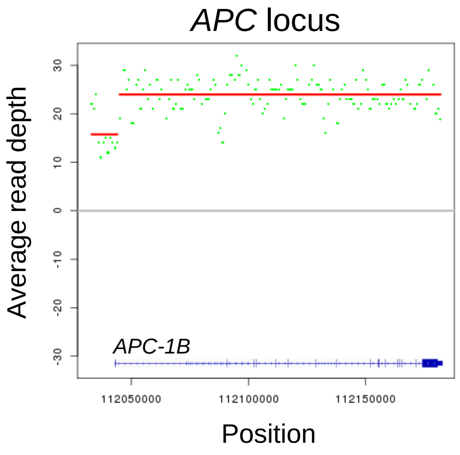

b

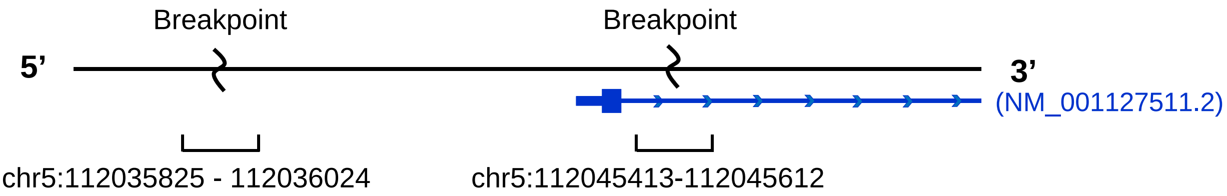


```
chr5_112035825-112036024_hg19_      CCAACATGGTGAAACCTTGTCACTACTGAAAA---TACAAAAATCAGCCG 47
chr5_112045413-112045612_hg19_      ---ACATGGCAAAGCCTCATCTGTACAAAAAAAAATACAAAAATAAGCCA 47
                                       ******  ** ***  **  ***  ****   ********* ****

chr5_112035825-112036024_hg19_      GGTGTGGTGGTGGGCGCTTGTAATCCCAGCTACTCGAGAGGCTGAGGCAG 97
chr5_112045413-112045612_hg19_      GGCATGGTGGTGCATGCCTGTGATTCCAGCTACTTGGGAGGCTGAGGCAG 97
                                    **  ********   ** *** ** ********* * *************

chr5_112035825-112036024_hg19_      GAGAATCGCTTGAACCCGAGAGGCGGAGGTTGCAGTGAGCCAAGATCGTG 147
chr5_112045413-112045612_hg19_      GAGGATTGTTGGAGCCCGGGATGTGGAGGTTGCAGTGAGCCAAGATTGTG 147
                                    *** ** * * ** **** ** * ********************** ***

chr5_112035825-112036024_hg19_      CCACTGCACTCCAGCCTGGGCGACAAGAGCAAAACTCCGTCTC--AAAAA 195
chr5_112045413-112045612_hg19_      CCATTGCACTCCAGTCTGGGTGACA---GTAAGACCCTGTCTCCTAACAA 194
                                    *** ********** ***** ****   * ** ** * *****  ** **

chr5_112035825-112036024_hg19_      AAAAA- 200
chr5_112045413-112045612_hg19_      ACAAAC 200
                                    * ***
```

Supplementary Figure S1. (**a**) Copy number analysis of the whole-genome sequence data using the circular binary segmentation algorithm with the R package DNAcopy. A significant reduction of copy number was detected in the 5’-flanking region of *APC-1B*. (**b**) A homology search of the flanking sequences of breakpoints. The DNA sequences (200 bp) encompassing the breakpoints were compared using The CLUSTALW (http://www.genome.jp/tools/clustalw/).

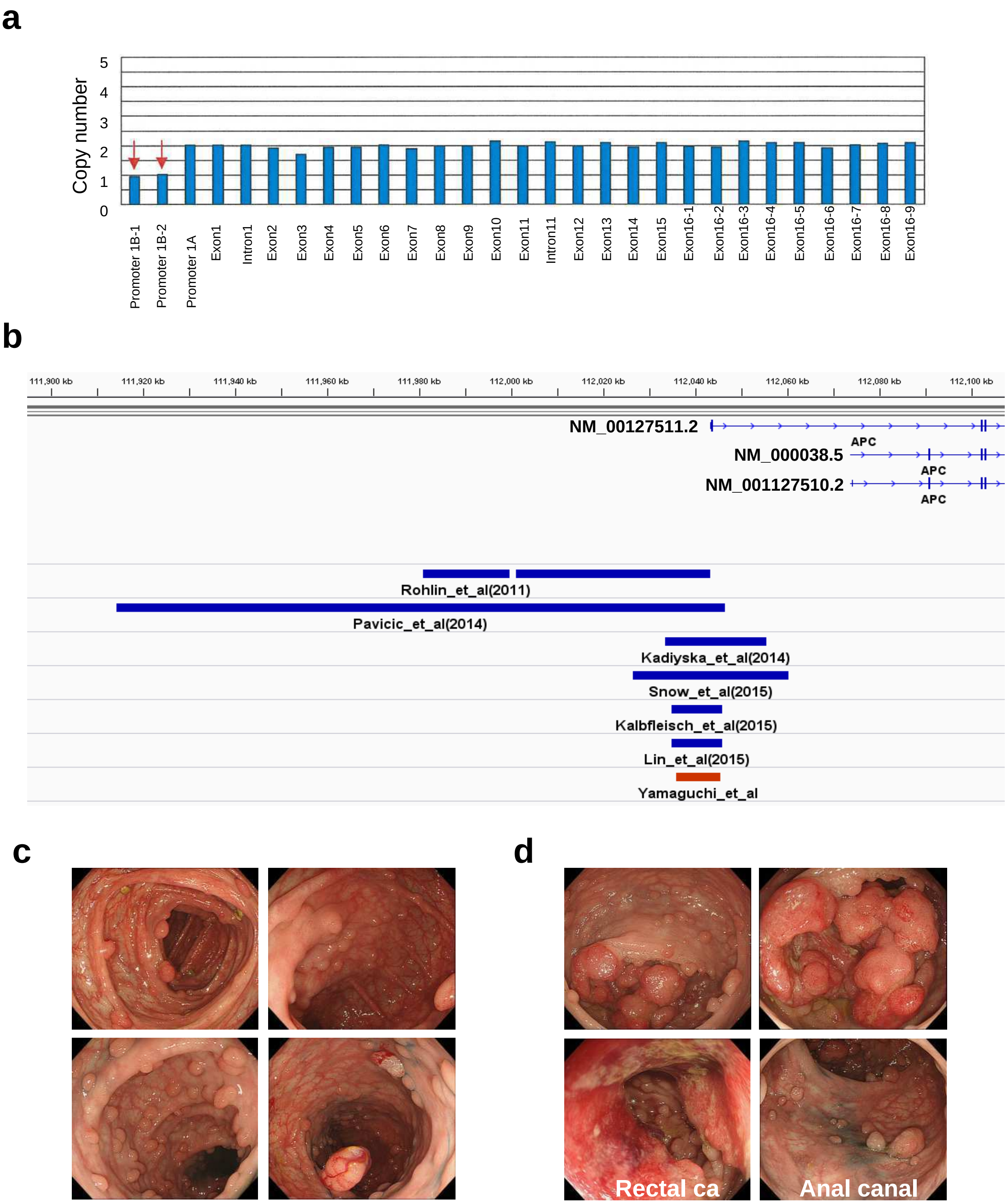

Supplementary Figure S2. (**a**) MLPA analysis of the patient. Twenty nine MLPA probes were designed to cover all exons of the *APC* gene (NM_000038.5) including promoter 1A and promoter 1B (NM_001127511.2), but do not contain any probes targeting the exon1 of the *APC-1B* variant (NM_00127511.2). This analysis corroborated the deletion of promoter 1B. No other copy number alterations were detected by the MLPA. (**b**) The reported deletions in the *APC* promoter 1B. The locations are shown according to GRCh37/hg19. The red bar represents the deletion identified in this study. Endoscopic images of the multiple colorectal polyps (**c**) and advanced rectal cancer (**d**).

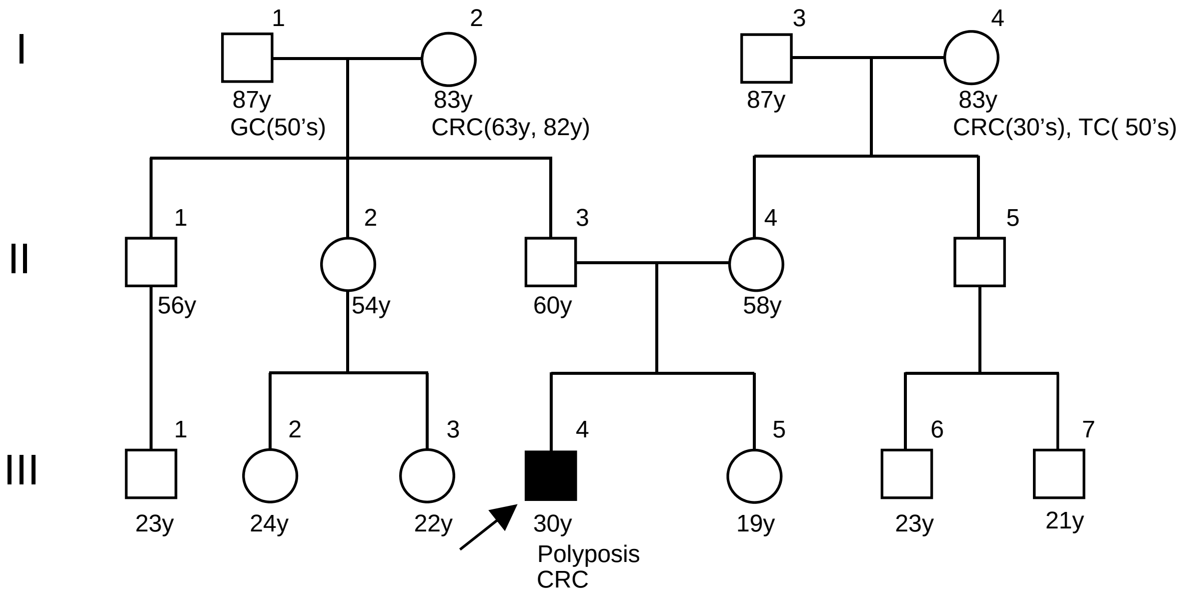

Supplementary Figure S3. The patient's family tree. The proband (patient) is indicated by an arrow. Males are illustrated by squares, and females by circles. Unaffected and affected individuals are represented by open and closed symbols, respectively. Current age and histories of malignancy are described under the symbols. GC; gastric cancer, CRC; colorectal cancer, TC; thyroid cancer. The age of disease onset is noted in the parentheses.

Supplementary Data S1 Enriched transcription factor binding motifs in the FAP patient using CAGE data (vs Control 1)

Case	Control	Up / Down	Motif No	Consensus	Foreground	Background	P-value	Known Motifs (P-value)
FAP	Control 1	Up	GLAM2_004	CNSCNSSCCCNSCNCCSCSBCBCNNNCCCGSCCCCBSCNCNCVCBNCNNC	155	5,594	3.42E-10	KROX(2.02756e-09),SP1:SP3(1.30064e-07),Sp1(2.58367e-07),Sp2(2.72344e-07),SP1(6.76489e-07), Sp4(7.03201e-07),Sp1(7.48223e-07),Sp1(9.40257e-07),SP4(1.66922e-06),SP1(6.53067e-06), Egr-1(6.62251e-06),Zfp281(1.14617e-05),Sp1(1.19267e-05),WT1(1.67534e-05),MAZ(1.96819e-05), Zfx(2.02042e-05),AP-2beta(2.96663e-05),CKROX(3.20863e-05),Zfp740(4.31589e-05),Egr1(6.73486e-05), CAC-binding_protein(8.87621e-05),Zfp410(9.5837e-05),MAZ(9.60922e-05)
FAP	Control 1	Up	GLAM2_004	CNSCNSSCCCNSCNCCSCSBCBCNNNCCCGSCCCCBSCNCNCVCBNCNNC	154	5,561	5.13E-10	KROX(2.02756e-09),SP1:SP3(1.30064e-07),Sp1(2.58367e-07),Sp2(2.72344e-07),SP1(6.76489e-07), Sp4(7.03201e-07),Sp1(7.48223e-07),Sp1(9.40257e-07),AP-2beta(2.96663e-05),SP4(1.66922e-06), SP1(6.53067e-06),Egr-1(6.62251e-06),Zfp281(1.14617e-05),Sp1(1.19267e-05),WT1(1.67534e-05), MAZ(1.96819e-05),Zfx(2.02042e-05),CKROX(3.20863e-05),Zfp740(4.31589e-05),Egr1(6.73486e-05), CAC-binding_protein(8.87621e-05),Zfp410(9.5837e-05),MAZ(9.60922e-05)
FAP	Control 1	Up	AMD_004	WAAWAAAAWAWAAWAAAAWAWAWNW	142	5,052	6.01E-09	FOXP1(1.1696e-05),Srf(5.40482e-05)
FAP	Control 1	Up	AMD_003	SNNNAGGNGTCNNTG	91	2,665	1.68E-08	Esrra(1.00361e-05)
FAP	Control 1	Up	DREME_004	AGGAAGTATTCA	73	2,031	1.70E-07	SPI1(7.37018e-07),ELF1(4.48042e-06),SPI1(7.86069e-06),C-ets-1(1.12708e-05)
FAP	Control 1	Up	AMD_005	WRAWCCAGCAGSNSMNDKNAA	94	3,282	3.14E-05	AP-4(6.50674e-05)
FAP	Control 1	Up	DREME_095	AAGGAAGTGCCTGG	67	2,099	4.00E-05	SPI1(1.27837e-05),c-Ets-1(2.17728e-05),ELF1(5.78363e-05),C-ets-1(6.81088e-05),SPI1(9.53238e-05)
FAP	Control 1	Up	AMD_008	GGWTCTAGGAANMTCCARCAGMNGG	75	2,447	4.18E-05	BCL6(1.88705e-05),Bcl-6(6.04164e-05)
FAP	Control 1	Up	DREME_094	AAGGAAGTGCCTGG	67	2,122	5.63E-05	SPI1(1.27837e-05),c-Ets-1(2.17728e-05),ELF1(5.78363e-05),C-ets-1(6.81088e-05),SPI1(9.53238e-05)
FAP	Control 1	Up	DREME_025	TATTACT	61	1,869	5.65E-05	NA
FAP	Control 1	Up	DREME_102	AAGTGTACTGGAGGA	58	1,752	6.34E-05	NA
FAP	Control 1	Up	DREME_147	AACACATGCACAT	75	2,544	1.53E-04	NA
FAP	Control 1	Up	DREME_056	AAGGAGATTTAG	47	1,365	1.66E-04	NA
FAP	Control 1	Up	DREME_003	ATCACACMCAC	54	1,651	1.71E-04	NA
FAP	Control 1	Up	DREME_122	ATTATGCAGG	45	1,294	1.91E-04	NA
FAP	Control 1	Up	DREME_111	ACAGTGCTGATCTC	51	1,536	1.94E-04	NA
FAP	Control 1	Up	DREME_026	AGTTTCATAT	52	1,593	2.47E-04	IRF-8(2.30024e-05),ICSBP(6.44783e-05)
FAP	Control 1	Up	DREME_018	ACTGTGCWCT	35	923	2.50E-04	NA
FAP	Control 1	Up	AMD_008	MNNNNTNMACCWKCAGNSNCW	73	2,496	2.56E-04	NA
FAP	Control 1	Up	DREME_039	AAGTGTACTGGAGGA	62	2,017	2.60E-04	NA
FAP	Control 1	Up	DREME_107	TAACACAGCTGA	57	1,810	2.81E-04	myogenin(4.53918e-06),Neuro_D(1.02953e-05)
FAP	Control 1	Up	DREME_081	AACCCAAACCTGA	75	2,594	2.85E-04	NA
FAP	Control 1	Up	DREME_154	ATGAAAYA	59	1,898	2.94E-04	NA
FAP	Control 1	Up	DREME_036	CTTAATAATC	44	1,287	3.30E-04	NA
FAP	Control 1	Up	DREME_023	ACGCAAGTGGG	29	717	3.36E-04	NA
FAP	Control 1	Up	DREME_041	AAGTGTACTGGAGGA	62	2,048	3.93E-04	NA
FAP	Control 1	Up	DREME_152	AATGTTCCATGA	36	1,012	6.40E-04	NA
FAP	Control 1	Up	DREME_158	AGCAGAATTTTA	73	2,589	7.69E-04	NA
FAP	Control 1	Up	DREME_008	AATATGTGGCT	47	1,473	8.73E-04	NA
FAP	Control 1	Up	DREME_032	AAAGGACAGCCTTT	61	2,073	9.28E-04	NA
FAP	Control 1	Up	DREME_062	AATATACCA	35	996	9.47E-04	NA
FAP	Control 1	Up	DREME_013	ATTTAGAAATA	101	3,930	1.06E-03	NA
FAP	Control 1	Up	DREME_136	AACTGCACATC	50	1,623	1.22E-03	NA
FAP	Control 1	Up	DREME_006	CTGGGTCCTGC	70	2,504	1.34E-03	CP2(5.55933e-05)
FAP	Control 1	Up	DREME_079	AAATAGCACTGG	56	1,889	1.39E-03	NA
FAP	Control 1	Up	DREME_113	AGGTTTGGGTTCCA	60	2,067	1.45E-03	NA
FAP	Control 1	Up	DREME_003	ATATCGTGTG	22	535	1.50E-03	NA
FAP	Control 1	Up	DREME_112	AGGTTTGGGTTCCA	60	2,080	1.69E-03	NA
FAP	Control 1	Up	DREME_002	ATATCGTGTG	22	545	1.87E-03	NA
FAP	Control 1	Up	DREME_124	ACCACGTAGAG	35	1,038	1.88E-03	NRSE(9.66024e-05)
FAP	Control 1	Up	DREME_019	AAGCATTCCCAAT	51	1,700	1.90E-03	Ik-1(8.07317e-05)
FAP	Control 1	Up	AMD_009	SNNTSYWGGARKNWCCARCNCCM	64	2,279	2.16E-03	NA
FAP	Control 1	Up	DREME_050	AAAGTCCCTCCCTG	52	1,757	2.26E-03	SP2(4.8283e-05),MAZ(4.84528e-05)
FAP	Control 1	Up	DREME_073	AAAGAAGGCCCA	51	1,716	2.31E-03	NA
FAP	Control 1	Up	DREME_095	AAGGAAGTGCCTG	53	1,803	2.34E-03	SPI1(1.02837e-05),c-Ets-1(3.57901e-05),ELF1(4.384e-05),C-ets-1(5.72639e-05),SPI1(7.57899e-05)
FAP	Control 1	Up	DREME_014	AAAGGACAGCCTTT	61	2,156	2.41E-03	NA
FAP	Control 1	Up	DREME_019	AGAACCATC	39	1,220	2.55E-03	NA
FAP	Control 1	Up	DREME_085	AAGACAGAGCTGGCAA	77	2,886	2.55E-03	NA
FAP	Control 1	Up	DREME_012	CATCAAGGGA	43	1,387	2.61E-03	NA
FAP	Control 1	Up	DREME_024	ACCATCTCTCAG	42	1,354	2.92E-03	NA
FAP	Control 1	Up	DREME_050	GGTCTCTGTCA	54	1,869	3.04E-03	NA
FAP	Control 1	Up	DREME_072	AAAGAAGGAAGTCCC	80	3,045	3.08E-03	SPI1(7.21674e-06),ELF1(3.38532e-05),C-ets-1(5.66411e-05),SPI1(6.66839e-05)
FAP	Control 1	Up	DREME_079	AAATTGAATGTATT	100	4,005	3.28E-03	NA
FAP	Control 1	Up	DREME_123	CTGTCTTCATTC	89	3,488	3.65E-03	NA
FAP	Control 1	Up	DREME_020	ACTCACTTCTG	71	2,648	3.68E-03	NA
FAP	Control 1	Up	DREME_067	AAAATTCAGATTG	84	3,253	3.71E-03	NA
FAP	Control 1	Up	DREME_129	ACCACGTAGAG	35	1,084	3.73E-03	NRSE(9.66024e-05)
FAP	Control 1	Up	DREME_030	CCTCATGAATC	33	1,004	3.76E-03	NA
FAP	Control 1	Up	DREME_111	CGACACCTCA	28	811	4.06E-03	Eomes(2.99327e-05)
FAP	Control 1	Up	DREME_133	CTGCTGGTTGAC	43	1,423	4.13E-03	NA
FAP	Control 1	Up	DREME_157	GATAGGAACC	43	1,426	4.28E-03	NA
FAP	Control 1	Up	DREME_047	GATGTTTATGC	41	1,343	4.34E-03	NA
FAP	Control 1	Up	DREME_087	AAGAGGAGACCAG	54	1,904	4.47E-03	NA
FAP	Control 1	Up	DREME_026	CCTCATGAATC	33	1,019	4.68E-03	NA
FAP	Control 1	Up	DREME_058	AATCCTTCCCTG	42	1,392	4.74E-03	NA
FAP	Control 1	Up	DREME_105	AATGGTCAGTAAGG	55	1,956	4.87E-03	NA
FAP	Control 1	Up	DREME_077	AAAGGACAGCCTTT	79	3,049	4.89E-03	NA
FAP	Control 1	Up	DREME_041	ATATATTTAATG	74	2,820	5.06E-03	NA
FAP	Control 1	Up	DREME_143	AAAGATTAGG	67	2,499	5.09E-03	NA
FAP	Control 1	Up	DREME_135	GGTGTTCCTTTA	57	2,066	6.06E-03	NA
FAP	Control 1	Up	DREME_125	CCACGGATA	14	317	6.10E-03	NA
FAP	Control 1	Up	DREME_155	AAATCTTCAA	74	2,842	6.17E-03	NA

FAP	Control 1	Up	DREME_033	ATGTGGATTCA	43	1,460	6.43E-03	NA
FAP	Control 1	Up	DREME_011	CAAACAATTG	61	2,255	6.66E-03	SOX17(7.61053e-05)
FAP	Control 1	Up	AMD_002	SCAYWCMCRATAYCTCATGAATCCA	57	2,079	6.90E-03	NA
FAP	Control 1	Up	DREME_002	ATATCGTGTG	25	727	6.90E-03	NA
FAP	Control 1	Up	DREME_124	AGGGTACAGAGCA	39	1,305	7.67E-03	NA
FAP	Control 1	Up	DREME_090	AAGCCACTCCGACA	29	904	9.06E-03	NA
FAP	Control 1	Up	DREME_077	GCGGCCGGCTGGA	33	1,071	9.46E-03	NA
FAP	Control 1	Up	DREME_093	AAGCCACTCCGACA	30	951	9.89E-03	NA
FAP	Control 1	Down	DREME_001	DTATAAA	120	2,115	3.92E-31	TBP(6.26328e-05)
FAP	Control 1	Down	AMD_003	AYANWNAYA	140	4,090	3.37E-16	NA
FAP	Control 1	Down	DREME_105	AATTGW	84	1,722	1.05E-15	NA
FAP	Control 1	Down	GLAM2_010	AARRAARARYARAAAANDAARM	178	6,372	1.26E-15	HNF3(9.02271e-06),Nanog(1.56317e-05),Zfp105(6.73915e-05),Sox11(7.5e-05)
FAP	Control 1	Down	AMD_001	TANNNNWA	139	4,153	4.46E-15	NA
FAP	Control 1	Down	AMD_006	WNWCYCHHHTNAAGTATASKYSADR	101	2,465	1.35E-14	NA
FAP	Control 1	Down	AMD_005	AWANNNCAT	142	4,468	1.40E-13	NA
FAP	Control 1	Down	AMD_002	ATNNNANW	134	4,077	1.97E-13	NA
FAP	Control 1	Down	DREME_005	ACTATATCTAT	90	2,145	3.52E-13	NA
FAP	Control 1	Down	DREME_053	AAACAA	124	3,641	5.71E-13	HFH4_(FOXJ1)(3.18859e-05),FOXO1(3.58716e-05),FOXO3(3.58716e-05),FOXO4(3.58716e-05), FOXO1(5.97853e-05),FOXP3(7.73072e-05),SRY(8.46249e-05),FOXO1(8.92187e-05),Foxk1(9.94188e-05)
FAP	Control 1	Down	DREME_082	AACTCTACTAT	81	1,861	2.01E-12	NA
FAP	Control 1	Down	DREME_010	ATACTTTA	50	808	2.05E-12	NA
FAP	Control 1	Down	DREME_026	TAAAAAATTTA	92	2,315	3.55E-12	NA
FAP	Control 1	Down	DREME_027	ATAKAAA	122	3,667	7.07E-12	NA
FAP	Control 1	Down	DREME_052	CATGATCCTA	48	795	1.44E-11	NA
FAP	Control 1	Down	DREME_004	ATAKAAA	122	3,724	2.21E-11	NA
FAP	Control 1	Down	DREME_072	ATCAGAC	34	423	2.63E-11	NA
FAP	Control 1	Down	DREME_024	AAAGTTGAACTAAG	102	2,876	8.34E-11	NA
FAP	Control 1	Down	DREME_034	AAAAAACCTTGTAAAG	122	3,815	1.27E-10	NA
FAP	Control 1	Down	DREME_043	AGAGAACCAAA	118	3,648	1.99E-10	NA
FAP	Control 1	Down	DREME_093	AATACCTCATT	87	2,310	3.76E-10	NA
FAP	Control 1	Down	DREME_126	AAAAATCCCAAA	123	3,940	5.21E-10	NA
FAP	Control 1	Down	DREME_132	CACTCAW	63	1,432	1.34E-09	NA
FAP	Control 1	Down	DREME_137	AAACTAAGAGTAG	88	2,424	1.79E-09	NA
FAP	Control 1	Down	DREME_115	TAATACA	75	1,900	1.91E-09	NA
FAP	Control 1	Down	DREME_001	ATAGATATAGTA	73	1,835	2.59E-09	NA
FAP	Control 1	Down	DREME_046	AAATTTMAGGG	96	2,788	2.79E-09	NA
FAP	Control 1	Down	DREME_002	ATAGATATAGTA	73	1,859	4.52E-09	NA
FAP	Control 1	Down	DREME_002	CAACTTACAC	51	1,054	4.59E-09	NA
FAP	Control 1	Down	DREME_016	ATGAATTAACTA	95	2,830	1.41E-08	NA
FAP	Control 1	Down	DREME_037	ATTTTGCTACA	84	2,360	1.46E-08	DMRT4(4.86777e-05)
FAP	Control 1	Down	DREME_044	GTTTRGGGCTA	49	1,030	1.70E-08	NA
FAP	Control 1	Down	DREME_106	TTACCTTAGA	74	1,977	2.52E-08	NA
FAP	Control 1	Down	DREME_035	AAAAATTATAAC	118	3,934	2.93E-08	NA
FAP	Control 1	Down	DREME_057	AATAACTTTG	80	2,237	3.44E-08	NA
FAP	Control 1	Down	AMD_003	TATKNNAAANNA	115	3,821	4.86E-08	Tcf7(6.14551e-06)
FAP	Control 1	Down	DREME_112	ATTHCA	94	2,863	5.64E-08	NA
FAP	Control 1	Down	AMD_006	RTMYCCTAAGTGTAWKYTKSKKRSW	73	1,993	8.02E-08	NA
FAP	Control 1	Down	DREME_144	AAGCTGTGRCT	53	1,232	9.13E-08	NA
FAP	Control 1	Down	DREME_156	AACTCAAAGGA	85	2,502	9.66E-08	LEF1(5.3009e-05),LEF-1(6.98162e-05)
FAP	Control 1	Down	AMD_007	RAKTRTARCYWARCATAAWRYAS	105	3,394	9.86E-08	NA
FAP	Control 1	Down	DREME_051	CTKCTAAATCC	54	1,279	1.19E-07	NA
FAP	Control 1	Down	DREME_161	GAAAGTGGCTTTA	94	2,911	1.29E-07	NA
FAP	Control 1	Down	DREME_021	TAAGTGTA	50	1,140	1.36E-07	NA
FAP	Control 1	Down	DREME_050	AAGAACAGCA	105	3,428	1.70E-07	NA
FAP	Control 1	Down	DREME_040	ATTCTRTCTTG	80	2,321	1.70E-07	NA
FAP	Control 1	Down	DREME_014	GTTTTAGTTAA	113	3,833	2.60E-07	HNF-1alpha(1.01232e-05),Tcf1(6.86962e-05)
FAP	Control 1	Down	AMD_002	YAMSMAMHHTACWCTTAGKWKAY	71	1,995	4.09E-07	NA
FAP	Control 1	Down	DREME_039	TAAGGTMA	54	1,331	4.11E-07	NA
FAP	Control 1	Down	AMD_009	THWNKGTTDTWTNATAGTARNRWK	120	4,212	4.59E-07	NA
FAP	Control 1	Down	DREME_061	ACAACACA	72	2,052	5.52E-07	FOXP1(7.83326e-05)
FAP	Control 1	Down	DREME_138	AGATGGAGTTTCA	98	3,184	5.80E-07	FOXM1(6.25326e-05),IRF-8(6.54402e-05)
FAP	Control 1	Down	DREME_123	ATTTACYCAAA	93	2,958	5.81E-07	NA
FAP	Control 1	Down	DREME_075	AAATAGCACACTC	66	1,826	7.74E-07	NA
FAP	Control 1	Down	AMD_004	TATKNNAAANNA	112	3,875	9.75E-07	Tcf7(7.07783e-06)
FAP	Control 1	Down	DREME_058	AACCTTGTCCT	72	2,093	1.16E-06	NA
FAP	Control 1	Down	DREME_006	ACAATTTCTAT	86	2,696	1.26E-06	NA
FAP	Control 1	Down	DREME_014	AAAATAGTGGGAAG	93	3,029	1.75E-06	RBP-Jkappa(4.18963e-05)
FAP	Control 1	Down	DREME_041	CAATTAAGAAA	115	4,065	1.83E-06	Nkx2-5(1.01983e-05),HOXB8(8.30317e-05),Nkx6-1(8.88834e-05)
FAP	Control 1	Down	DREME_055	ACAGTAATA	60	1,646	2.42E-06	NA
FAP	Control 1	Down	DREME_007	TCAACTCAA	53	1,382	2.86E-06	NA
FAP	Control 1	Down	DREME_060	CCAAGATTACA	59	1,631	3.87E-06	NA
FAP	Control 1	Down	DREME_020	ACACTTACCAT	62	1,760	4.57E-06	NA
FAP	Control 1	Down	DREME_008	AAGAACAGC	89	2,924	5.34E-06	NA
FAP	Control 1	Down	DREME_018	TCACCCTCCTCA	101	3,492	6.77E-06	NA
FAP	Control 1	Down	DREME_135	ATCCTTCCCTGAC	52	1,392	7.62E-06	NA
FAP	Control 1	Down	DREME_050	AAGAACAGCAT	86	2,816	7.85E-06	AR(9.84114e-05)
FAP	Control 1	Down	DREME_145	AGGCGATA	19	279	7.98E-06	NA
FAP	Control 1	Down	DREME_003	GAGTGTAGCTT	52	1,396	8.25E-06	NA
FAP	Control 1	Down	DREME_078	AAACACACAGCCATC	77	2,424	8.31E-06	NA
FAP	Control 1	Down	DREME_078	TAAGGAAAAGAAA	145	5,726	8.55E-06	NA
FAP	Control 1	Down	DREME_027	CATGATCCTM	52	1,401	9.10E-06	NA
FAP	Control 1	Down	DREME_080	AAATAGCACACT	52	1,402	9.28E-06	NA

FAP	Control 1	Down	DREME_043	AGATTCTATCTTG	77	2,433	9.53E-06	NA
FAP	Control 1	Down	DREME_024	AACATGGTAAGTGT	68	2,052	9.93E-06	NA
FAP	Control 1	Down	AMD_009	WTTAMCTAGAAATAWCTTTKCA	113	4,098	1.03E-05	NA
FAP	Control 1	Down	DREME_012	GAAATTGTAA	78	2,486	1.10E-05	NA
FAP	Control 1	Down	DREME_051	CAATGGTAAT	62	1,811	1.12E-05	NA
FAP	Control 1	Down	AMD_001	GAAAWAACYTTGYAAAGAGARYMA	113	4,110	1.20E-05	NA
FAP	Control 1	Down	DREME_088	ATCCTTCCCTGAC	52	1,422	1.36E-05	NA
FAP	Control 1	Down	AMD_001	GAAAWAACYTTGYAAAGAGARYMA	113	4,124	1.43E-05	NA
FAP	Control 1	Down	DREME_114	ATYTTCTTCAGC	93	3,184	1.59E-05	NA
FAP	Control 1	Down	DREME_007	ATGAATTAACTA	73	2,301	1.77E-05	NA
FAP	Control 1	Down	DREME_012	AAAAAATTTAATA	97	3,388	2.04E-05	NA
FAP	Control 1	Down	DREME_027	AACATAAAGCACC	68	2,100	2.13E-05	NA
FAP	Control 1	Down	DREME_005	AGAACAGCTA	60	1,774	2.39E-05	NA
FAP	Control 1	Down	DREME_084	AACTGAGGTTGGA	79	2,612	3.58E-05	NA
FAP	Control 1	Down	DREME_056	CAGGGAACTT	67	2,093	3.66E-05	NA
FAP	Control 1	Down	DREME_104	ACTGGCTGCAATG	37	906	3.71E-05	NA
FAP	Control 1	Down	DREME_040	AAAGAGGAACAGCTC	85	2,888	3.96E-05	NA
FAP	Control 1	Down	DREME_004	AGAACAGCTA	60	1,809	4.23E-05	NA
FAP	Control 1	Down	DREME_072	AAAATATTAAACT	86	2,945	4.62E-05	NA
FAP	Control 1	Down	DREME_110	TCTGTAGGHA	61	1,859	4.83E-05	NA
FAP	Control 1	Down	DREME_153	ACTGAGCAGTA	53	1,549	6.45E-05	NA
FAP	Control 1	Down	DREME_077	CACAACTCCCAC	64	2,009	6.97E-05	NA
FAP	Control 1	Down	DREME_006	AGCTACACTC	47	1,318	7.37E-05	NA
FAP	Control 1	Down	DREME_127	AGGTGGATTTAG	58	1,776	9.21E-05	NA
FAP	Control 1	Down	DREME_081	AACCTCAGCTGCA	50	1,451	9.41E-05	myogenin(9.62468e-06),Neuro_D(1.95772e-05),LBP-1(2.39138e-05)
FAP	Control 1	Down	DREME_112	AACTCAAGAACTG	70	2,288	9.47E-05	NA
FAP	Control 1	Down	DREME_051	AAAACAATTGCA	90	3,196	1.08E-04	NA
FAP	Control 1	Down	DREME_012	AGAAAGCGTTC	47	1,341	1.10E-04	NA
FAP	Control 1	Down	DREME_164	CAGATGACC	32	779	1.22E-04	NA
FAP	Control 1	Down	AMD_006	AAWWHTAATACMMAWAKTAGSYCKR	112	4,259	1.25E-04	NA
FAP	Control 1	Down	DREME_049	CACAACTCCCAC	64	2,060	1.45E-04	NA
FAP	Control 1	Down	DREME_009	ATGAATTAACTA	76	2,594	1.64E-04	NA
FAP	Control 1	Down	DREME_046	AAACCTAGCCCYA	56	1,737	1.79E-04	TRF1(3.55082e-05)
FAP	Control 1	Down	DREME_048	AACCTTGTCCT	57	1,785	1.96E-04	NA
FAP	Control 1	Down	DREME_015	TAGTGTCCAAA	50	1,500	2.08E-04	NA
FAP	Control 1	Down	DREME_018	AAAGAGCTGTTCC	63	2,044	2.10E-04	ZBRK1(2.41616e-05)
FAP	Control 1	Down	DREME_134	ATCCTAACGG	30	733	2.17E-04	NA
FAP	Control 1	Down	DREME_017	AAGCTACACTCT	47	1,383	2.23E-04	NA
FAP	Control 1	Down	DREME_142	CACTCTGAG	42	1,188	2.35E-04	NA
FAP	Control 1	Down	DREME_041	AGGATCATG	48	1,427	2.37E-04	NA
FAP	Control 1	Down	GLAM2_007	RVARRRARAAARAAVARARA	175	7,733	2.40E-04	Srf(7.29793e-05)
FAP	Control 1	Down	GLAM2_007	RVARRRARAAARAAVARARA	175	7,733	2.40E-04	Srf(7.29793e-05)
FAP	Control 1	Down	DREME_050	GCAGTGWCTTA	58	1,841	2.40E-04	Gfi1b(4.0975e-05)
FAP	Control 1	Down	DREME_143	CGCGCGCAATA	14	222	2.68E-04	NA
FAP	Control 1	Down	DREME_167	AAAGCTCTCCCTC	67	2,236	2.70E-04	NA
FAP	Control 1	Down	DREME_087	ATAGTGAGTGC	36	967	2.82E-04	NA
FAP	Control 1	Down	DREME_065	ACTCAGACTCTG	49	1,479	2.83E-04	NA
FAP	Control 1	Down	DREME_040	AGCAGTGTCTTA	42	1,199	2.84E-04	NA
FAP	Control 1	Down	DREME_006	AACAGTTAAAT	89	3,235	2.90E-04	NA
FAP	Control 1	Down	DREME_125	TTCTTCTGAGW	91	3,335	3.13E-04	NA
FAP	Control 1	Down	DREME_133	AGAGACATGGAA	93	3,430	3.16E-04	NA
FAP	Control 1	Down	DREME_104	AAAACAAAAGGCTGG	119	4,701	3.38E-04	NA
FAP	Control 1	Down	DREME_100	ACGCGCGCAATA	24	544	3.55E-04	NA
FAP	Control 1	Down	DREME_094	ACAAATGTAAG	88	3,207	3.57E-04	NA
FAP	Control 1	Down	DREME_097	TCTAAGGTAA	29	721	3.66E-04	NA
FAP	Control 1	Down	AMD_005	AAWWHTAATACMMAWAKTAGSYCKR	110	4,262	3.67E-04	NA
FAP	Control 1	Down	DREME_028	ACCCGTCTAT	13	202	3.70E-04	NA
FAP	Control 1	Down	DREME_110	AAATGGTTTGGTTAA	80	2,842	3.70E-04	NA
FAP	Control 1	Down	DREME_017	AAAATTTAATAC	90	3,308	3.88E-04	NA
FAP	Control 1	Down	DREME_062	CTGGCACTGTAC	42	1,219	3.98E-04	NA
FAP	Control 1	Down	DREME_119	ATCTTAGAAGAGA	90	3,311	4.01E-04	NA
FAP	Control 1	Down	DREME_038	GGTTTTAGTTA	56	1,795	4.10E-04	NA
FAP	Control 1	Down	DREME_142	CCGAAAGTAGG	44	1,304	4.33E-04	NA
FAP	Control 1	Down	DREME_105	ACTTTCCCTGCGC	65	2,187	4.40E-04	NA
FAP	Control 1	Down	DREME_023	GTGGCTGCTTTTA	74	2,586	4.43E-04	NA
FAP	Control 1	Down	DREME_139	CTCCTACTGTC	60	1,972	4.48E-04	NA
FAP	Control 1	Down	DREME_092	ACTGTCCTTCCAG	56	1,802	4.52E-04	NA
FAP	Control 1	Down	DREME_073	CAGACTCAGCA	71	2,461	4.93E-04	MAF(2.64355e-05),Nrf-2(3.25401e-05),NF-E2(8.88945e-05)
FAP	Control 1	Down	DREME_067	AAGAGTGTGCT	46	1,395	5.08E-04	NA
FAP	Control 1	Down	DREME_044	AGCAGTGTCTTA	42	1,240	5.59E-04	NA
FAP	Control 1	Down	DREME_125	AACTGACAATT	55	1,786	6.44E-04	NA
FAP	Control 1	Down	AMD_008	THWRMTKTTAGKYCWAMKWKRAR	72	2,532	6.70E-04	NA
FAP	Control 1	Down	DREME_032	CCACCTTACTA	30	787	6.72E-04	NA
FAP	Control 1	Down	DREME_108	ATAGGTGTTG	41	1,215	7.09E-04	MyoD(2.93833e-05)
FAP	Control 1	Down	DREME_067	ATCCACTGGTA	37	1,061	7.60E-04	NA
FAP	Control 1	Down	DREME_129	AAATTTAAGGGT	46	1,426	8.04E-04	NA
FAP	Control 1	Down	DREME_026	ACACTAGGAAAAA	103	3,999	8.10E-04	Helios_A(5.97834e-05),NF-AT4(9.74814e-05)
FAP	Control 1	Down	AMD_008	KMWRAACTRANGTTRGDAKNAK	88	3,287	8.40E-04	NA
FAP	Control 1	Down	DREME_060	CTGGCACTGTAC	42	1,271	9.05E-04	NA
FAP	Control 1	Down	DREME_025	AACTCAACTTGAC	48	1,518	9.18E-04	NA
FAP	Control 1	Down	DREME_020	ACCCGTCTAT	13	227	1.01E-03	NA
FAP	Control 1	Down	DREME_103	CTGGGGAGGAA	65	2,257	1.02E-03	MZF1(1.26122e-05)

FAP	Control 1	Down	DREME_032	AGTAAGGTGGA	41	1,239	1.03E-03	NA
FAP	Control 1	Down	DREME_077	AAAAGAAAGCTGAA	115	4,621	1.09E-03	NA
FAP	Control 1	Down	DREME_095	ACACTACTTGCA	41	1,244	1.11E-03	NA
FAP	Control 1	Down	DREME_141	CTGGGGAGGAA	65	2,265	1.12E-03	MZF1(1.26122e-05)
FAP	Control 1	Down	DREME_129	AGATGTTACA	54	1,787	1.13E-03	NA
FAP	Control 1	Down	AMD_004	CCTCTATATAAATGCGYAGGGGT	50	1,620	1.18E-03	NA
FAP	Control 1	Down	DREME_148	AAAAGGACTGAG	89	3,368	1.18E-03	NA
FAP	Control 1	Down	DREME_150	CTCAGCAGCTGCC	58	1,963	1.19E-03	Ascl2(1.66561e-06),myogenin(1.71293e-05),LBP-1(2.77213e-05),myogenin(3.05709e-05), Neuro_D(3.14007e-05),MATH1(6.23993e-05),MAFA(6.40561e-05)
FAP	Control 1	Down	DREME_042	AATCTCCTAA	56	1,883	1.29E-03	NA
FAP	Control 1	Down	DREME_086	AACATGAGCTCCG	35	1,015	1.30E-03	NA
FAP	Control 1	Down	DREME_083	AAGCCTACATCAG	42	1,298	1.35E-03	NA
FAP	Control 1	Down	DREME_022	AAAGTGCACTTGGA	48	1,551	1.44E-03	NA
FAP	Control 1	Down	DREME_126	CTGGGGAGGAA	65	2,295	1.58E-03	MZF1(1.26122e-05)
FAP	Control 1	Down	DREME_073	ATAATAYAGCAAG	62	2,164	1.61E-03	NA
FAP	Control 1	Down	DREME_132	ACCTTGTAACC	33	955	1.76E-03	NA
FAP	Control 1	Down	DREME_063	AATATTAAAAAC	87	3,317	1.81E-03	NA
FAP	Control 1	Down	DREME_094	AATACTAATAAG	70	2,532	1.81E-03	NA
FAP	Control 1	Down	DREME_119	AGGTGGATCAC	41	1,280	1.87E-03	NA
FAP	Control 1	Down	DREME_053	ATTTTCTTCAGC	91	3,513	1.93E-03	NA
FAP	Control 1	Down	DREME_111	AACGAAGTCAGTCAC	48	1,576	1.99E-03	NA
FAP	Control 1	Down	DREME_030	AAATAAAGTATAG	89	3,422	2.00E-03	NA
FAP	Control 1	Down	DREME_084	AAGTAACATGAA	53	1,794	2.10E-03	NA
FAP	Control 1	Down	DREME_019	AAAGCGTTCAA	57	1,969	2.13E-03	NA
FAP	Control 1	Down	DREME_087	AACGAAGTCAGTCAC	48	1,582	2.14E-03	NA
FAP	Control 1	Down	DREME_072	AACCTTGTCCT	48	1,583	2.17E-03	NA
FAP	Control 1	Down	DREME_052	AGCTGCAACA	59	2,064	2.32E-03	NA
FAP	Control 1	Down	DREME_155	CTCAGCAGCTGCC	56	1,938	2.47E-03	Ascl2(1.66561e-06),myogenin(1.71293e-05),LBP-1(2.77213e-05),myogenin(3.05709e-05), Neuro_D(3.14007e-05),MATH1(6.23993e-05),MAFA(6.40561e-05)
FAP	Control 1	Down	DREME_069	GAAAACAATTGC	66	2,383	2.51E-03	NA
FAP	Control 1	Down	DREME_116	CTCTGTAGTCAC	32	938	2.52E-03	NA
FAP	Control 1	Down	DREME_054	CTGACWCATC	28	783	2.53E-03	AP-1(2.03938e-05),Fra-1(3.54518e-05),AP-1(6.20096e-05),AP1(6.59113e-05),FRA1(8.23479e-05)
FAP	Control 1	Down	DREME_124	AAAAAGATCCT	75	2,804	2.82E-03	NA
FAP	Control 1	Down	DREME_077	CTCTCTCAGACA	45	1,477	2.82E-03	NA
FAP	Control 1	Down	DREME_018	GCTTGGTTATA	42	1,363	3.29E-03	NA
FAP	Control 1	Down	DREME_011	CTTCCCACTA	49	1,660	3.30E-03	RBP-Jkappa(1.73951e-05)
FAP	Control 1	Down	DREME_129	ACAGACTCTA	29	840	3.45E-03	NA
FAP	Control 1	Down	DREME_102	ACTCCCTGAGTAG	31	919	3.47E-03	NA
FAP	Control 1	Down	DREME_085	AATACTAATAAG	70	2,597	3.50E-03	NA
FAP	Control 1	Down	DREME_033	AGGGCCCTGAA	38	1,202	3.50E-03	NA
FAP	Control 1	Down	DREME_119	ACAAATGTAAGTGT	59	2,104	3.58E-03	NA
FAP	Control 1	Down	DREME_151	ATCCTTCCCCA	68	2,509	3.61E-03	ZBP89(9.63684e-05)
FAP	Control 1	Down	DREME_071	AGCATATGG	37	1,166	3.75E-03	NA
FAP	Control 1	Down	DREME_045	AGTATACTTCAA	33	1,005	3.82E-03	NA
FAP	Control 1	Down	DREME_054	AACTGCCCATAT	45	1,502	3.85E-03	NA
FAP	Control 1	Down	DREME_019	AAAGAGGAACAGCT	75	2,841	4.01E-03	NA
FAP	Control 1	Down	DREME_079	ACTTTGCCTTC	68	2,524	4.18E-03	NA
FAP	Control 1	Down	DREME_131	AATGCCCCTGCCT	66	2,437	4.34E-03	NA
FAP	Control 1	Down	DREME_070	AACGAAGTCAG	47	1,603	4.63E-03	NA
FAP	Control 1	Down	DREME_041	ACTTTGCCTTC	68	2,538	4.78E-03	NA
FAP	Control 1	Down	DREME_114	AACTGTGGTGCA	33	1,024	5.03E-03	AML1(2.38821e-05),AML1(2.85909e-05),AML1a(5.77709e-05)
FAP	Control 1	Down	DREME_112	ATTTTCTTCAGC	91	3,623	5.13E-03	NA
FAP	Control 1	Down	DREME_013	CCACCTTACTA	46	1,570	5.19E-03	NA
FAP	Control 1	Down	DREME_118	ATCCCTCTGGCTG	59	2,143	5.35E-03	NA
FAP	Control 1	Down	DREME_166	CTCTGGCTACTC	49	1,706	5.58E-03	NA
FAP	Control 1	Down	DREME_042	AAATGGTTTGGTTA	61	2,240	5.76E-03	NA
FAP	Control 1	Down	DREME_011	GTGCACTTTCCA	50	1,756	5.99E-03	NF-kappaB(8.74193e-05)
FAP	Control 1	Down	DREME_162	GAAGATGTGTC	49	1,714	6.09E-03	NA
FAP	Control 1	Down	DREME_036	ACGTGCAGTG	17	423	6.34E-03	HIF-1alpha(9.14916e-05)
FAP	Control 1	Down	DREME_097	AGGGAGTCACAAC	38	1,248	6.38E-03	NA
FAP	Control 1	Down	DREME_091	AGAGCAGCTGCGA	64	2,387	6.42E-03	myogenin(1.45604e-05),Ascl2(1.55872e-05),LBP-1(2.11245e-05),Neuro_D(3.05948e-05),HEN1(4.8765e-05) HEN1(7.96414e-05),myogenin(9.67472e-05)
FAP	Control 1	Down	DREME_131	ATAGGTGTTG	44	1,503	6.45E-03	MyoD(2.93833e-05)
FAP	Control 1	Down	DREME_122	GGGTGCCATAC	17	425	6.62E-03	NA
FAP	Control 1	Down	DREME_029	AAAATCCCAAAC	89	3,563	6.91E-03	NA
FAP	Control 1	Down	DREME_082	AAACACACAGCCATC	74	2,855	6.92E-03	NA
FAP	Control 1	Down	DREME_021	AAAGTATAGGCG	42	1,424	6.95E-03	NA
FAP	Control 1	Down	DREME_104	ACTGGGATCCTGC	49	1,729	7.16E-03	NA
FAP	Control 1	Down	DREME_106	CTTAGCCSTA	30	928	7.19E-03	NA
FAP	Control 1	Down	DREME_035	CACCAAGCAAC	47	1,646	7.49E-03	NA
FAP	Control 1	Down	DREME_097	ACAAATGTAAGTG	70	2,681	7.65E-03	NA
FAP	Control 1	Down	DREME_017	GGTTCGTCCAA	28	852	7.65E-03	NA
FAP	Control 1	Down	DREME_049	CACTGCACGTGC	36	1,181	7.85E-03	HIF1A:ARNT(7.68297e-05),HIF-1alpha(8.42317e-05)
FAP	Control 1	Down	DREME_015	AAAATCCCAAACA	104	4,308	7.88E-03	NA
FAP	Control 1	Down	DREME_150	AAGGTCTGTG	56	2,048	7.89E-03	NA
FAP	Control 1	Down	DREME_089	AACTTCAGTCTT	49	1,740	8.04E-03	NA
FAP	Control 1	Down	DREME_143	TGAGCTGGYAA	49	1,744	8.38E-03	NA
FAP	Control 1	Down	DREME_016	AAACCAGACGAGCT	53	1,922	8.51E-03	NA
FAP	Control 1	Down	DREME_065	GGTTGCCTCCA	29	903	8.93E-03	NA
FAP	Control 1	Down	DREME_037	CCATAGYAGGC	22	627	8.99E-03	NA
FAP	Control 1	Down	DREME_079	AAAAGGTCCCTA	56	2,062	9.04E-03	NA
FAP	Control 1	Down	DREME_111	CCGGTTTACAA	27	824	9.11E-03	NA

FAP	Control 1	Down	DREME_096	ACCCGCAGACTCAG	56	2,066	9.39E-03	NA
FAP	Control 1	Down	DREME_031	ACAGGGGTTAGT	40	1,365	9.41E-03	NA
FAP	Control 1	Down	DREME_147	GGGCTGCACTCA	60	2,251	9.80E-03	NA

Supplementary Data S1 Enriched transcription factor binding motifs in the FAP patient using CAGE data (vs Control 2)

Case	Control	Up / Down	Motif No	Consensus	Foreground	Background	P-value	Known Motifs (P-value)
FAP	Control 2	Up	DREME_073	ATGAACTATG	62	1,449	5.44E-09	NA
FAP	Control 2	Up	DREME_081	CTGMTCACA	56	1,307	3.90E-08	NA
FAP	Control 2	Up	DREME_034	AAGAGGAGACCAG	73	2,159	1.78E-06	NA
FAP	Control 2	Up	DREME_148	CCCCATACAAA	59	1,627	3.59E-06	NA
FAP	Control 2	Up	DREME_030	CCTCATGAATC	53	1,399	4.05E-06	NA
FAP	Control 2	Up	DREME_161	ATGAACATC	50	1,301	5.70E-06	NA
FAP	Control 2	Up	DREME_020	ACCAACACAC	56	1,535	5.93E-06	NA
FAP	Control 2	Up	DREME_045	AAGCATTCCCAAT	69	2,083	8.31E-06	Ik-1(8.07317e-05)
FAP	Control 2	Up	DREME_037	AAAGGACAGCCTT	81	2,607	9.44E-06	NA
FAP	Control 2	Up	DREME_026	AAGAGGAGACCAG	77	2,435	9.83E-06	NA
FAP	Control 2	Up	DREME_025	AAGAGGAGACCAG	77	2,439	1.04E-05	NA
FAP	Control 2	Up	DREME_015	ATATCGTGTG	35	785	1.10E-05	NA
FAP	Control 2	Up	DREME_024	CCATGGACTGA	44	1,118	1.39E-05	NA
FAP	Control 2	Up	DREME_126	TTCTGAAGAAA	98	3,412	1.51E-05	NA
FAP	Control 2	Up	DREME_003	ATAAAAACAATA	131	5,033	1.52E-05	NA
FAP	Control 2	Up	AMD_009	RTATAWNWNANWCWTWNTDNWW	120	4,479	1.61E-05	NA
FAP	Control 2	Up	AMD_006	RMRBGTACAKAKNACCTGADDSYK	64	1,920	1.75E-05	NA
FAP	Control 2	Up	DREME_095	AAACTGGAACAA	72	2,283	2.57E-05	DMRT5(2.65954e-05),DMRT7(8.68454e-05)
FAP	Control 2	Up	DREME_155	ACAGAGCCACATA	64	1,967	3.69E-05	NA
FAP	Control 2	Up	DREME_016	CCTCATGAATC	39	983	4.06E-05	NA
FAP	Control 2	Up	DREME_082	GGGTGATTTAC	42	1,101	4.62E-05	NA
FAP	Control 2	Up	DREME_056	AATACATTCAAT	83	2,813	4.84E-05	NA
FAP	Control 2	Up	GLAM2_007	GVDNVDGGSNGGGGGNGGGGNGGGGGVKV	176	7,664	4.84E-05	MAZ(2.57113e-08),SP1(5.14095e-08),Zfp281(4.03971e-07),Zfp281(7.48165e-07),Sp1(3.03981e-06), Zfp740(4.82357e-06),SP1(7.67453e-06),ZNF219(8.08973e-06),KROX(8.36362e-06),ZBP89(1.04717e-05), CAC-binding_protein(1.4667e-05),Sp1(1.83673e-05),SP1:SP3(3.11156e-05),Sp2(3.37051e-05), Sp1(3.90655e-05),MAZ(4.42019e-05),SP4(4.6235e-05),CKROX(4.95634e-05), FPM315_(ZNF263)(6.23779e-05),Ascl2(7.98853e-05),PLAG1(9.11055e-05),WT1(9.5828e-05)
FAP	Control 2	Up	DREME_098	AAAGACTGAAGATG	79	2,639	5.17E-05	NA
FAP	Control 2	Up	GLAM2_007	GVDNVDGGSNGGGGGNGGGGNGGGGGVKV	176	7,678	5.78E-05	MAZ(2.57113e-08),SP1(5.14095e-08),Zfp281(4.03971e-07),Zfp281(7.48165e-07),Sp1(3.03981e-06), Zfp740(4.82357e-06),SP1(7.67453e-06),ZNF219(8.08973e-06),KROX(8.36362e-06),ZBP89(1.04717e-05), CAC-binding_protein(1.4667e-05),Sp1(1.83673e-05),SP1:SP3(3.11156e-05),Sp2(3.37051e-05), Sp1(3.90655e-05),MAZ(4.42019e-05),SP4(4.6235e-05),CKROX(4.95634e-05), FPM315_(ZNF263)(6.23779e-05),Ascl2(7.98853e-05),PLAG1(9.11055e-05),WT1(9.5828e-05)
FAP	Control 2	Up	DREME_092	AAGGCAGACCCCA	72	2,344	6.17E-05	LF-A1(8.92976e-05)
FAP	Control 2	Up	DREME_068	AGATGTACAT	53	1,553	6.89E-05	NA
FAP	Control 2	Up	DREME_091	GAGAAAGATTA	98	3,543	7.80E-05	NA
FAP	Control 2	Up	DREME_088	ATTCCAAGTA	57	1,730	8.58E-05	NA
FAP	Control 2	Up	DREME_060	ACAGGACATGTG	34	836	8.74E-05	NA
FAP	Control 2	Up	DREME_083	CTCTGTAACCA	62	1,944	9.29E-05	NA
FAP	Control 2	Up	DREME_070	GAGTCATGTCA	47	1,338	1.05E-04	C-Jun(2.50016e-05)
FAP	Control 2	Up	DREME_062	ATATCCACCGAG	24	500	1.09E-04	NA
FAP	Control 2	Up	DREME_067	GCCTGAACACCA	53	1,590	1.25E-04	NA
FAP	Control 2	Up	DREME_149	AAGGCAGGGTCTC	72	2,405	1.41E-04	NA
FAP	Control 2	Up	DREME_128	AGCCCTCACTGA	49	1,439	1.50E-04	NA
FAP	Control 2	Up	DREME_031	AAATTGAATGTATT	81	2,815	1.57E-04	NA
FAP	Control 2	Up	DREME_134	AGCCAGCCTG	59	1,865	1.85E-04	NA
FAP	Control 2	Up	DREME_115	AATAGCATGTGC	42	1,178	1.97E-04	NA
FAP	Control 2	Up	DREME_004	AGTTTCATAT	53	1,634	2.47E-04	ICSBP(8.50572e-05)
FAP	Control 2	Up	DREME_103	AACATCACCACAT	67	2,248	3.15E-04	AML1(1.067e-05),AML1(1.49156e-05),AML1a(4.23846e-05)
FAP	Control 2	Up	DREME_122	ACATCAAAATAG	89	3,245	3.25E-04	NA
FAP	Control 2	Up	DREME_097	ACATCAAAATAG	89	3,248	3.36E-04	NA
FAP	Control 2	Up	DREME_093	AATAGCATGTGCA	50	1,534	3.51E-04	NA
FAP	Control 2	Up	DREME_019	CAGTCCATG	28	684	3.57E-04	NA
FAP	Control 2	Up	DREME_093	AGAGGATCTCCT	62	2,041	3.59E-04	NA
FAP	Control 2	Up	DREME_063	CCAAATGAGGAG	50	1,544	4.07E-04	SRF(3.27352e-05),SRF(6.86455e-05),SRF(7.46369e-05),SRF(8.73253e-05)
FAP	Control 2	Up	DREME_066	CCAAATGAGGAG	50	1,550	4.44E-04	SRF(3.27352e-05),SRF(6.86455e-05),SRF(7.46369e-05),SRF(8.73253e-05)
FAP	Control 2	Up	DREME_147	CAATTCTAGAA	72	2,500	4.61E-04	NA
FAP	Control 2	Up	AMD_009	GKRNATRYNNNNCTGNCC	54	1,733	5.58E-04	PUR1(2.49845e-05)
FAP	Control 2	Up	DREME_010	GAGTCATGTCA	35	967	5.71E-04	C-Jun(2.70895e-05)
FAP	Control 2	Up	DREME_013	AAAATTCAGATT	101	3,879	6.43E-04	NA
FAP	Control 2	Up	DREME_092	TGTAATCAAAA	71	2,486	6.61E-04	NA
FAP	Control 2	Up	DREME_132	CTGCATTCAA	54	1,746	6.65E-04	NA
FAP	Control 2	Up	DREME_059	CACATGTCCTGTC	41	1,212	6.76E-04	NA
FAP	Control 2	Up	DREME_038	AAGCATTCCCAATA	57	1,876	6.86E-04	Ik-3(3.55236e-05),Ik-1(7.97252e-05)
FAP	Control 2	Up	DREME_114	GATATTGCA	36	1,017	6.98E-04	NA
FAP	Control 2	Up	DREME_044	AACTGCTGGATGT	63	2,139	7.16E-04	NA
FAP	Control 2	Up	DREME_073	AAACACTTGTGAC	56	1,839	7.39E-04	NA
FAP	Control 2	Up	DREME_136	AAAGAACAGCCC	61	2,065	8.42E-04	NA
FAP	Control 2	Up	DREME_083	AAAGACTGAAGA	82	3,012	8.78E-04	NA
FAP	Control 2	Up	DREME_139	ATATTAWG	34	956	9.28E-04	FOXJ2(9.53668e-05)
FAP	Control 2	Up	DREME_135	GATATTGCA	31	842	9.39E-04	NA
FAP	Control 2	Up	DREME_047	ACCATCTCTCAGC	43	1,315	9.53E-04	NA
FAP	Control 2	Up	DREME_101	ACAGCTGTAAATG	57	1,902	9.55E-04	myogenin(1.92494e-06),Neuro_D(3.91547e-06)
FAP	Control 2	Up	DREME_005	ATCACACCCAC	31	843	9.57E-04	NA
FAP	Control 2	Up	DREME_012	ACACGATAT	36	1,037	9.79E-04	NA
FAP	Control 2	Up	DREME_091	AGAGGATCTCCT	62	2,122	9.89E-04	NA
FAP	Control 2	Up	DREME_040	AAATCTCCTTCTCT	81	2,981	1.03E-03	NA
FAP	Control 2	Up	DREME_045	AAGTGTACTGGAG	45	1,406	1.09E-03	NA
FAP	Control 2	Up	DREME_004	AAAAACAATAAAAA	142	6,013	1.10E-03	FOXP1(1.29021e-05),Sox7(5.01042e-05),Sox8(5.16543e-05),SOX17(6.09698e-05),SRY(7.65906e-05)
FAP	Control 2	Up	DREME_138	AAAGAACAGCCC	61	2,098	1.25E-03	NA

FAP	Control 2	Up	DREME_037	AAGAATTATTG	66	2,321	1.28E-03	NA
FAP	Control 2	Up	DREME_019	ACCAACACAC	57	1,926	1.28E-03	NA
FAP	Control 2	Up	DREME_060	TCCCTATCAGCA	33	938	1.32E-03	NA
FAP	Control 2	Up	DREME_046	ACAGTGCTGATC	32	901	1.36E-03	NA
FAP	Control 2	Up	DREME_101	AAATTTAACGTC	63	2,196	1.40E-03	NA
FAP	Control 2	Up	DREME_018	ACAAGCTTTGCAG	54	1,809	1.49E-03	NA
FAP	Control 2	Up	DREME_094	AAAAGGACATC	66	2,344	1.66E-03	NA
FAP	Control 2	Up	DREME_163	GAAACCAGGGCCA	61	2,123	1.67E-03	NA
FAP	Control 2	Up	DREME_069	CCAAATGAGGAG	62	2,170	1.73E-03	SRF(4.29099e-05),SRF(8.8072e-05)
FAP	Control 2	Up	DREME_053	AGCCGGCCATGCA	26	688	1.77E-03	NA
FAP	Control 2	Up	DREME_049	ACAGGACATGTG	37	1,115	1.80E-03	NA
FAP	Control 2	Up	DREME_117	AATGACCTCAGC	35	1,036	1.82E-03	PPARA(6.51455e-05),LXRalpha:RXRalpha(8.84002e-05),T3RALPHA(9.20762e-05)
FAP	Control 2	Up	DREME_096	AATGACCTCAGC	39	1,209	2.18E-03	PPARA(6.51455e-05),LXRalpha:RXRalpha(8.84002e-05),T3RALPHA(9.20762e-05)
FAP	Control 2	Up	DREME_099	AAAGTTGGATAA	65	2,329	2.28E-03	NA
FAP	Control 2	Up	DREME_135	AAACTAAAGCACC	67	2,431	2.59E-03	NA
FAP	Control 2	Up	DREME_139	AAAGGCAATGACTC	50	1,691	2.87E-03	NA
FAP	Control 2	Up	DREME_035	AACTGCTGGATGT	51	1,740	3.07E-03	NA
FAP	Control 2	Up	DREME_038	AAAGTCATCTGG	53	1,839	3.54E-03	NA
FAP	Control 2	Up	DREME_017	ACCAACACAC	57	2,017	3.64E-03	NA
FAP	Control 2	Up	DREME_042	AACCTGGCAGCAATG	40	1,289	3.71E-03	NA
FAP	Control 2	Up	DREME_014	GTGAGCCGGTTA	26	731	3.87E-03	NA
FAP	Control 2	Up	DREME_112	AAGGCAGACCCCAG	65	2,381	3.90E-03	NA
FAP	Control 2	Up	DREME_066	ACCACATAGT	25	694	3.95E-03	AML1(2.08863e-06),AML1(3.45239e-06),AML1a(1.36919e-05),Osf2(9.86483e-05)
FAP	Control 2	Up	DREME_096	AAAGACAATTT	77	2,938	4.17E-03	NA
FAP	Control 2	Up	DREME_081	AAATTTAACGTC	70	2,617	4.24E-03	NA
FAP	Control 2	Up	DREME_184	GGCACATCCCA	28	816	4.40E-03	NA
FAP	Control 2	Up	DREME_121	GGGTGATTTAC	29	856	4.45E-03	NA
FAP	Control 2	Up	DREME_003	CCCGTGTCACTG	47	1,600	4.47E-03	NA
FAP	Control 2	Up	DREME_011	ATGGTTCTAGGA	49	1,691	4.72E-03	BCL6(4.61905e-06)
FAP	Control 2	Up	DREME_051	AGCATGTGGATTC	44	1,480	4.93E-03	NA
FAP	Control 2	Up	DREME_032	AAATTGAATGTA	68	2,542	4.96E-03	NA
FAP	Control 2	Up	DREME_087	AAATTTAACGTC	70	2,643	5.42E-03	NA
FAP	Control 2	Up	DREME_180	CGAGCACAGCC	36	1,154	5.55E-03	NA
FAP	Control 2	Up	DREME_010	CCCGTGTCACTG	47	1,619	5.56E-03	NA
FAP	Control 2	Up	DREME_061	ACCATCTCTCAG	34	1,077	5.95E-03	NA
FAP	Control 2	Up	DREME_118	AAACCYGAGCCTG	61	2,244	5.99E-03	NA
FAP	Control 2	Up	DREME_061	ACCACATAGT	25	719	6.06E-03	AML1(2.08863e-06),AML1(3.45239e-06),AML1a(1.36919e-05),Osf2(9.86483e-05)
FAP	Control 2	Up	DREME_107	GAATGAAACAGC	47	1,628	6.15E-03	NA
FAP	Control 2	Up	DREME_053	AGAGAGTTCAGAG	68	2,565	6.16E-03	NA
FAP	Control 2	Up	DREME_076	AAACACTTGTGACTG	61	2,247	6.16E-03	NA
FAP	Control 2	Up	DREME_017	GTGAGCCGGTTA	26	760	6.27E-03	NA
FAP	Control 2	Up	DREME_036	AAACAGAAGTTT	89	3,554	6.42E-03	NA
FAP	Control 2	Up	DREME_041	AAATCTTTGTGA	71	2,711	6.62E-03	NA
FAP	Control 2	Up	DREME_008	GGAACATCACA	46	1,593	6.74E-03	NA
FAP	Control 2	Up	DREME_133	AAGTATTCAT	56	2,036	7.01E-03	NA
FAP	Control 2	Up	DREME_130	GGGTGATTTAC	29	888	7.21E-03	NA
FAP	Control 2	Up	DREME_002	AAGCAGAAACCCT	77	3,003	7.42E-03	NA
FAP	Control 2	Up	DREME_033	AACCTGGCAGCAA	43	1,473	7.47E-03	NA
FAP	Control 2	Up	DREME_097	AAGAAGAATTAT	92	3,723	7.86E-03	NA
FAP	Control 2	Up	DREME_053	AGCAAAGAAACA	94	3,820	7.89E-03	NA
FAP	Control 2	Up	DREME_063	AGAGAGTTCAGAG	78	3,058	7.94E-03	NA
FAP	Control 2	Up	DREME_105	AACCGTACTA	14	328	8.01E-03	NA
FAP	Control 2	Up	DREME_089	AACCGTACTAT	25	737	8.09E-03	NA
FAP	Control 2	Up	AMD_008	WNACAANNNNWKCANWNT	103	4,265	8.27E-03	NA
FAP	Control 2	Up	DREME_154	ACTGAAGACAGAG	72	2,785	8.45E-03	NA
FAP	Control 2	Up	DREME_035	AACTGCTGGATGT	51	1,833	8.47E-03	NA
FAP	Control 2	Up	DREME_115	AACTGCATGGGC	53	1,927	8.94E-03	NA
FAP	Control 2	Up	DREME_029	AGCCGGCCATGC	39	1,320	9.15E-03	NA
FAP	Control 2	Up	DREME_151	AACAGGGTGT	40	1,364	9.30E-03	KLF3(1.5272e-05),AR(6.85588e-05)
FAP	Control 2	Down	AMD_008	ATGTNSNNTGANRW	91	2,246	1.74E-12	NA
FAP	Control 2	Down	DREME_003	TCAGDAA	107	2,977	6.58E-12	NA
FAP	Control 2	Down	AMD_006	KNRCWGNNNACA	91	2,310	8.48E-12	NA
FAP	Control 2	Down	AMD_009	MNNDNAAGNNRKTTC	116	3,483	5.04E-11	NA
FAP	Control 2	Down	AMD_005	AGABHTTGNNNSM	98	2,705	8.75E-11	NA
FAP	Control 2	Down	AMD_002	AWGNNNCTG	91	2,516	8.77E-10	NA
FAP	Control 2	Down	DREME_011	CASTCAA	63	1,427	1.17E-09	NA
FAP	Control 2	Down	DREME_008	CASTCAA	63	1,445	1.89E-09	NA
FAP	Control 2	Down	DREME_002	TTATRA	62	1,410	1.96E-09	NA
FAP	Control 2	Down	AMD_004	CAGRWMACA	109	3,460	1.34E-08	NA
FAP	Control 2	Down	AMD_003	WTAWNNNAAANW	137	4,976	1.08E-07	Mtf1(2.28822e-05)
FAP	Control 2	Down	DREME_005	CACAGGA	61	1,547	1.39E-07	NA
FAP	Control 2	Down	DREME_018	TGAATR	62	1,613	2.53E-07	Sox8(6.21761e-05),Sox17(8.29006e-05)
FAP	Control 2	Down	AMD_003	ACANATCYNNNW	92	2,914	5.88E-07	NA
FAP	Control 2	Down	AMD_003	AGANANRNTCANNR	123	4,379	5.96E-07	NA
FAP	Control 2	Down	AMD_002	CAGRAGACA	103	3,417	6.05E-07	NA
FAP	Control 2	Down	AMD_002	ACANATCYNNNW	91	2,936	1.67E-06	NA
FAP	Control 2	Down	DREME_009	AGATHAA	79	2,426	2.19E-06	NA
FAP	Control 2	Down	AMD_006	AWGNVYCTGNNY	73	2,212	4.35E-06	NA
FAP	Control 2	Down	AMD_006	WWANNNNATT	123	4,581	8.84E-06	Dbx-1(4.3375e-05)
FAP	Control 2	Down	DREME_020	CATGCAAACAA	69	2,179	3.70E-05	NA
FAP	Control 2	Down	AMD_004	RNNNATGHNNCAK	59	1,771	4.45E-05	NA
FAP	Control 2	Down	GLAM2_009	RGGARGRRRRRGNARGRRGARR	183	8,115	4.73E-05	Gabpa(9.36917e-06),PU.1(1.41052e-05)

FAP	Control 2	Down	AMD_005	WDNGATGNNTGGT	65	2,073	9.52E-05	NA
FAP	Control 2	Down	AMD_007	WDNGATGNNTGGT	65	2,098	1.35E-04	NA
FAP	Control 2	Down	DREME_014	AGATGAGGA	91	3,343	3.41E-04	NA
FAP	Control 2	Down	DREME_005	CCTCAAACCACAG	65	2,189	4.51E-04	AML1(5.15324e-06),AML1(6.75259e-06),core-binding_factor(9.74253e-06),AML1a(1.25949e-05), AML1(1.79198e-05),AML3(4.19039e-05),RUNX1(7.28083e-05)
FAP	Control 2	Down	DREME_022	GGTGAATGTCA	40	1,173	6.85E-04	NA
FAP	Control 2	Down	AMD_004	AANNWCTTRTGA	92	3,546	1.68E-03	NA
FAP	Control 2	Down	DREME_007	GGTTCTTTGAA	57	1,955	1.81E-03	NA
FAP	Control 2	Down	DREME_019	GGTGAATGTCA	40	1,237	1.82E-03	NA
FAP	Control 2	Down	AMD_008	ACTCACCNAGGAS	71	2,583	1.92E-03	NA
FAP	Control 2	Down	DREME_007	AGTAATCCGG	16	344	2.14E-03	NA
FAP	Control 2	Down	AMD_005	WAWKAW	92	3,579	2.28E-03	NA
FAP	Control 2	Down	DREME_004	ATTCTGGGGTG	55	1,890	2.35E-03	SREBP-1(3.66178e-05)
FAP	Control 2	Down	AMD_007	ACTCACCNAGGAS	71	2,611	2.56E-03	NA
FAP	Control 2	Down	DREME_001	AGTAATCCGG	16	351	2.58E-03	NA
FAP	Control 2	Down	DREME_003	ACCCCAGAATG	54	1,877	3.32E-03	NA
FAP	Control 2	Down	AMD_001	KGGTNNGNWTACM	60	2,189	5.42E-03	NA
FAP	Control 2	Down	AMD_009	WMNNATCNWNNWNTAA	113	4,785	9.91E-03	NA
